# The motility and chemosensory systems of *Rhizobium leguminosarum*, their role in symbiosis, and link to PTS^Ntr^ regulation

**DOI:** 10.1111/1462-2920.16570

**Published:** 2024-01-12

**Authors:** Samuel T. N. Aroney, Francesco Pini, Celia Kessler, Philip S. Poole, Carmen Sánchez-Cañizares

**Affiliations:** Department of Biology, University of Oxford, Oxford, UK

## Abstract

Motility and chemotaxis are crucial processes for soil bacteria and plant–microbe interactions. This applies to the symbiotic bacterium *Rhizobium leguminosarum*, where motility is driven by flagella rotation controlled by two chemotaxis systems, Che1 and Che2. The Che1 cluster is particularly important in free-living motility prior to the establishment of the symbiosis, with a *che1* mutant delayed in nodulation and reduced in nodulation competitiveness. The Che2 system alters bacteroid development and nodule maturation. In this work, we also identified 27 putative chemoreceptors encoded in the *R. leguminosarum bv. viciae* 3841 genome and characterized its motility in different growth conditions. We describe a metabolism-based taxis system in rhizobia that acts at high concentrations of dicarboxylates to halt motility independent of chemotaxis. Finally, we show how PTS^Ntr^ influences cell motility, with PTS^Ntr^ mutants exhibiting reduced swimming in different media. Motility is restored by the active forms of the PTS^Ntr^ output regulatory proteins, unphosphorylated ManX and phosphorylated PtsN. Overall, this work shows how rhizobia typify soil bacteria by having a high number of chemoreceptors and highlights the importance of the motility and chemotaxis mechanisms in a free-living cell in the rhizosphere, and at different stages of the symbiosis.

## Introduction

Soil is a highly competitive environment for microbial life, with as many as 10^4^ bacterial species and 10^9^ bacterial cells per gram (Poole et al., 2018). Within the soil, the rhizosphere and the root surface, specialized colonizing bacteria are selected by plants through secretion of phytochemicals and proteins from their roots (Badri & Vivanco, 2009; Rodríguez-Navarro et al., 2007; Tkacz et al., 2020; Wheatley & Poole, 2018). In the case of legumes, this leads to the recruitment of endophytic symbionts and the formation of root nodules (Oldroyd & Downie, 2008; Poole et al., 2018). A molecular dialogue between rhizobia and legume plants leads to root hair curling, forcing the bacteria inside the root where they form infection threads (Fisher & Long, 1992; Poole et al., 2018). Bacterial movement through infection threads is mainly due to continual growth and cell division, but the filling of infection threads is not continuous, with bacteria reaching the tip of the thread before the tube is filled, suggesting another form of motility is also used (Fournier et al., 2008; Gage, 2004). Within the root, rhizobia bacteria reach prepared nodule cells where these bacteria differentiate into nitrogen-fixing bacteroids (Monahan-Giovanelli et al., 2006; Poole et al., 2018). At nodule senescence, undifferentiated bacteria or non-terminally differentiated bacteroids from either indeterminate or determinate nodules, respectively, are released back into the rhizosphere to start the symbiosis cycle again (Gage, 2004; Oldroyd & Downie, 2008; Wheatley et al., 2020).

Most symbiotic bacteria selected from the environment have motility and chemotaxis systems (Raina et al., 2019). These systems are key to the establishment of successful symbioses, being necessary for competitive nodulation (Catlow et al., 1990; Mendoza-Suárez et al., 2020; Miller et al., 2007; Yost et al., 1998), and plant root surface colonization (Scharf et al., 2016). A bacterium can achieve a growth advantage by arriving and establishing itself at the root surface before a competitive environment has formed (Allard-massicotte et al., 2016). In addition, flagella-based swarming motility enables movement along the surface of the root for more effective colonization (Gao et al., 2016; Simons et al., 1996).

*Rhizobium leguminosarum* bv. *viciae* 3841 (Rlv3841) is an α-proteobacterium and a well-established model for symbiotic nitrogen fixation, nodulating pea (*Pisum sativum*) and vetch (*Vicia villosa*) (Johnston & Beringer, 1975; Poole et al., 1994). Rlv3841 motility systems include flagella, and two chemotaxis systems encoded in the chromosome: Che1 (RL0686 to RL0693, *icpAcheX1Y1A1W1R1B1Y1D1*), class F7, and Che2 (RL4036 to RL4028, *cheY3A2W2mcrCBA-cheW3R2B2*), class F8 (Aroney et al., 2021; Scharf et al., 2016; Wuichet & Zhulin, 2010; Young et al., 2006) (Figure S1). The flagella and main chemotaxis operon, *che1*, is orthologous to the well-studied chemotaxis operon controlling flagellar motility in *Sinorhizobium meliloti* (Arapov et al., 2020; Attmannspacher et al., 2005; Scharf et al., 2001; Sourjik & Schmitt, 1998). The second chemotaxis system, *che2*, is orthologous to genes coding for one of the chemotaxis pathways involved in controlling flagellar motility in *Rhodobacter sphaeroides* (Hamblin et al., 1997; Miller et al., 2007). Notably, two model rhizobia were found to down-regulate genes related to motility and chemotaxis in nodules despite the genes being essential for nodule bacteria and bacteroid recovery in an insertion sequencing (INSeq) mutagenesis (Becker et al., 2004; Karunakaran et al., 2009, 2010; Tambalo et al., 2010; Wheatley et al., 2020; Yost et al., 2004). INSeq mutants in the *che2* cluster had reduced performance in nodule bacteria (i.e., undifferentiated cells regrown from nodules) but were neutral in all other categories, suggesting that a functional *che2* cluster results in increased differentiation.

Rlv3841 flagellar and chemotaxis genes are transcriptionally activated by the heterodimer VisNR and Rem (Rotter et al., 2006; Sourjik et al., 2000; Tambalo et al., 2010). Interestingly, acting by protein–protein interactions, the phosphotransferase system (PTS) of *Vibrio fischeri* and the paralogous nitrogen-metabolism regulator PTS^Ntr^ of *Escherichia coli* has been found to control motility, through EIIA^Glc/Ntr^ interacting with flagella formation or chemotaxis (Gravina et al., 2021; Park et al., 2016; Visick et al., 2007). In α-proteobacteria, such as Rlv3841, the PTS system is restricted to only the nitrogen-PTS (PTS^Ntr^) encoded by the genes *ptsP, npr* and *ptsN* (EIIA^Ntr^) and the remaining component of the carbohydrate-PTS system, *manX* (EIIA^Man^). PTS are signal transduction pathways that regulate various cellular functions by sensing the availability of nitrogen and carbohydrates in the environment and accordingly balancing central metabolism. We have previously shown that Rlv3841 has a single PTS^Ntr^ (Sánchez-Cañizares et al., 2020), but it is currently unknown whether this system influences motility.

In this study, we clarify the roles of motility and chemotaxis in free-living cells and during the different symbiotic stages. Firstly, we categorized the chemotaxis receptors of Rlv3841. We demonstrate that high concentrations of TCA-cycle intermediates (dicarboxylates) halt motility via metabolism-based taxis independent of the Che systems and show how PTS^Ntr^ influences swimming motility. We confirm the importance of flagella and the Che1 cluster for free-living motility and competitive nodulation and show that the Che2 cluster is important for bacteroid development and nodule maturation.

## Experimental Procedures

### Bacterial strains, plasmids and growth conditions

The bacterial strains, plasmids, and oligonucleotides that were used in this study are listed in Tables S2, S3, and S4. *E. coli* strains were grown at 37°C in Luria-Bertani (LB) medium (Sambrook et al., 1989) with or without 1.4% agar supplemented with appropriate antibiotics μg · mL^–1^: kanamycin, 20; ampicillin, 100; spectinomycin, 50 and tetracycline, 10. In addition, X-gal (5-bromo-4-chloro-3-indolyl-*β*-D-galactopyranoside) at 40 mg/mL was added to media for blue-white screening with *E. coli. R. leguminosarum* strains were grown at 28°C in Tryptone-yeast (TY) medium (Beringer, 1974), and Universal Minimal Salts (UMS) (Wheatley et al., 2016) with appropriate carbon and nitrogen sources as stated in each experiment, with or without 1.75% agar supplemented with appropriate antibiotics as above, ug/mL: kanamycin (40 standard, 160 for pK19mobSacB selection); streptomycin, 500; spectinomycin, 100; tetracycline (5 in agar, 1 in liquid). Plasmids were introduced into *E. coli* by heat shock at 42°C and into *R. leguminosarum* by biparental or triparental mating (Figurski & Helinski, 1979; Thoma & Schobert, 2009).

### Generation of stable mutants and tagged strains

Mutants were generated using the pK18mobSacB vector for stable double recombinants, and marking was done with (Choi & Schweizer, 2006) mini-Tn7 integration (see Supporting Information for details).

### Semi-solid agar swimming assay

To assess the swimming ability of strains under various conditions, they were grown on 0.22% agar UMS media plates supplemented with 10 mM glucose and 10 mM ammonium, unless otherwise stated. Inoculants were grown on TY slopes for 3 days before being resuspended in 3–4 mL UMS and normalized to an optical density of OD_600_ = 1.8. The plates were incubated at 28°C for 4–7 days before the bacterial halos were measured in mm.

### Single-cell motility tracking

To assess the distribution of swimming patterns within strain populations, individual cells were tracked, following a capillaray assay adapted from Baker et al. (2016). Each strain was grown on TY slopes for 3 days before being resuspended in 3–4 mL liquid UMS cultures and diluted to an optical density of OD600 = 0.1 in 10 mL of UMS supplemented with 10 mM glucose and 10 mM NH_4_Cl. These cultures were incubated overnight at 28°C. Next, the cultures were diluted again to an optical density of OD600 = 0.1 in 10 mL of UMS supplemented with the tested carbon and nitrogen sources. These cultures were incubated for 3 h at 28°C and staggered to keep the incubation time constant. Each culture was then drawn into a 0.2-by-2-mm glass capillary tube by capillary action, sealed at both ends with vacuum grease and placed on a microscope slide. The cells were then visualized on a Meiji microscope with a 40 × lens and recorded using an Infinity lite camera (1440 × 1080 pixels at 10 fps). Four technical replicate images were taken along the length of the capillary, at the top surface of the capillary. The images were then processed through an image analysis pipeline (github.com/AroneyS/Capillary-tracking) based on (Oliveira et al., 2022). This pipeline produced individual tracks for each bacterium, calculating the overall swimming speed and the tumble rate.

### Plant growth conditions

Plants were grown as by Mendoza-Suárez et al. (2020) with the following specifications. Seeds of *Pisum sativum* cv. Aveola (pea) and *Vicia villosa* (vetch) were surface sterilized and germinated on distilled water agar plates as above. Seedlings planted in pots were filled with either a 50:50 mixture of medium vermiculite and silver sand, or only medium vermiculite, or in boiling tubes filled with fine vermiculite supplemented with nitrogen-free rooting solution (Poole et al., 1994). Plants were grown in a controlled environment room (random positioning) at 21°C, 16/8 h a day/night cycle. The germinated vetch seedlings were typically grown on filter paper in 10 cm square Petri dishes containing 1% agar FP media sealed with Millipore tape in a controlled environment room with the aforementioned conditions (Fahraeus, 1957; Pini et al., 2017).

### Nodule competition assay

To differentiate nodules colonized by different strains on the same plant, the bacteria were marked with *gusA* and *celB* to enable differential staining with Magenta-GlcA and X-Gal, respectively (Sánchez-Cañizares & Palacios, 2013). Tagged bacteria were then grown on TY slopes for 3 days. Peas were germinated and two peas per 500 mL pot were planted within a mix of 50/50 fine vermiculite and silver sand as above. A 10^5^ 1:1 mix of the bacterial strains for comparison was added to each pot. The bacterial mixes were also plated in triplicate on TY plates supplemented with X-glcA (5-bromo-4-chloro-3-indolyl-*β*-D-glucuronic acid CHA salt) to confirm the mixture ratios. Negative controls were made by adding water. The plants were harvested after 21 days post-inoculation (dpi) and stained sequentially and nodules were then counted by colour to determine the output ratio of strains.

### Nodule development assay

Pea plants were grown in pots with sand:vermiculite pots as above and individually inoculated with 1 mL of 10^5^ cells of Rlv3841 *celB* marked strains. The plants were harvested after 14, 21 and 28 dpi, and the roots were weighted and stained as above. The roots were subsequently photographed and analysed using an image analysis pipeline (github.com/AroneyS/Nodule-segmentation).

### Nodulation dynamics assay

*Vicia villosa* (hairy vetch) plants were grown on square plates with FP agar as above and Rlv3841 strains harbouring plasmid pLMB712 (*pnodA::lux* promoter fusion) were individually inoculated with 50 μL of 10^5^ cells directly along each root. Plates were imaged daily using NightOWL In Vivo Imaging System, recording luminescence spikes and first nodule appearance.

### Bacteria and bacteroid isolation from mature nodules

This assay was used to determine the proportion of undifferentiated bacteria and bacteroids in the nodules formed by various Rlv3841 strains. Peas were germinated and planted in 500 mL pots of fine vermiculite with 1 mL 10^5^ inoculum of constitutive sfGFP-marked bacteria added to each pot. After 21 days, 10 mature nodules per plant were harvested, weighed and crushed into 1 mL of bacteroid isolation buffer (Green et al., 2019). The mixture was then centrifuged at 1000 rpm for 10 min to pellet plant debris. The supernatant was recovered and centrifuged again at 6000 rpm for 5 min, with the resulting cell pellet resuspended in 1 mL 0.9% NaCl. This wash was repeated once. The cell sample was then diluted 1:10 in flow-cytometer grade PBS for subsequent flow cytometer analysis. Samples were analysed by measuring forward and side scatter alongside fluorescence until 5000 reasonable events were counted. Constitutive sfGFP labelled cells were distinguished by setting a threshold of fluorescence with further gates differentiating bacteria and bacteroids based on forward and size scatter. The bacteroids are larger (forward scatter) and more granular (side scatter) than the undifferentiated bacteria.

### Data-handling and statistical analysis

Data handling, statistical analysis and graph generation were carried out in R version 4.0.3 (R Core Team, 2019). Statistical analysis was performed using the packages lme4 1.1.26 (Bates et al., 2015), lmerTest 3.1.3 (Kuznetsova et al., 2017) and Emmeans 1.5.4 (Lenth, 2020).

## Results

### Rlv3841 encodes a variety of chemoreceptors

The Rlv3841 genome consists of seven replicons: a large chromosome and six plasmids (Young et al., 2006). We searched all Rlv3841 genes for the highly conserved methyl-accepting chemotaxis proteins (MCP) signal domain (CheA/CheW binding region) and identified 27 candidate chemoreceptors (Table S1). Chemoreceptor genes were distributed across the entire genome, with 21 in the main chromosome, one in pRL8, one in pRL10 and four in pRL12. Twenty-five out of the 27 putative MCPs had sensor domains, whereas the other two encoded only the methylation domain. Notably, one chemoreceptor was located within the *che1* cluster in the chromosome (RL0685, *icpA*), and three were located within the *che2* cluster in the chromosome (RL4031-4033, *mcrABC*). We aligned the Rlv3841 chemoreceptor protein sequences to eight chemoreceptors from the well-studied *S. meliloti* RU11/001 to generate a phylogenetic tree (Figure S2). In addition to the MCP signal domain, most chemore-ceptors contained one or more of the following domains: HAMP domains, acting as signal transmission from sensor domain to the signalling domain (Salah Ud-Din & Roujeinikova, 2017); sensory domains, highly varied and including a variety of classes (e.g., Cache, PAS, protoglobin); transmembrane domains, typically present in a pair and surrounding the sensory domain, predicting that most sensory domains are located in the periplasm, with the remaining sensors being detached from the membrane and sensing in the cytoplasm (see Figure S3). All Rlv3841 chemoreceptors are class 36H (consisting of 36 heptads, with a structure highly conserved, see Figure S4A), except McrA, McrB and McrC (within the *che2* cluster) which are class 34H (Alexander & Zhulin, 2007; Ortega & Zhulin, 2018). Most of the Rlv3841 chemoreceptors closely resemble the classic methylation sequences of *E. coli* Tsr (Figure S4B,D) (Rice & Dahlquist, 1991) with some alterations conserved within Rlv3841 (Figure S4C,E).

### General motility properties of Rlv3841 in free-living conditions

**Figure 1:**
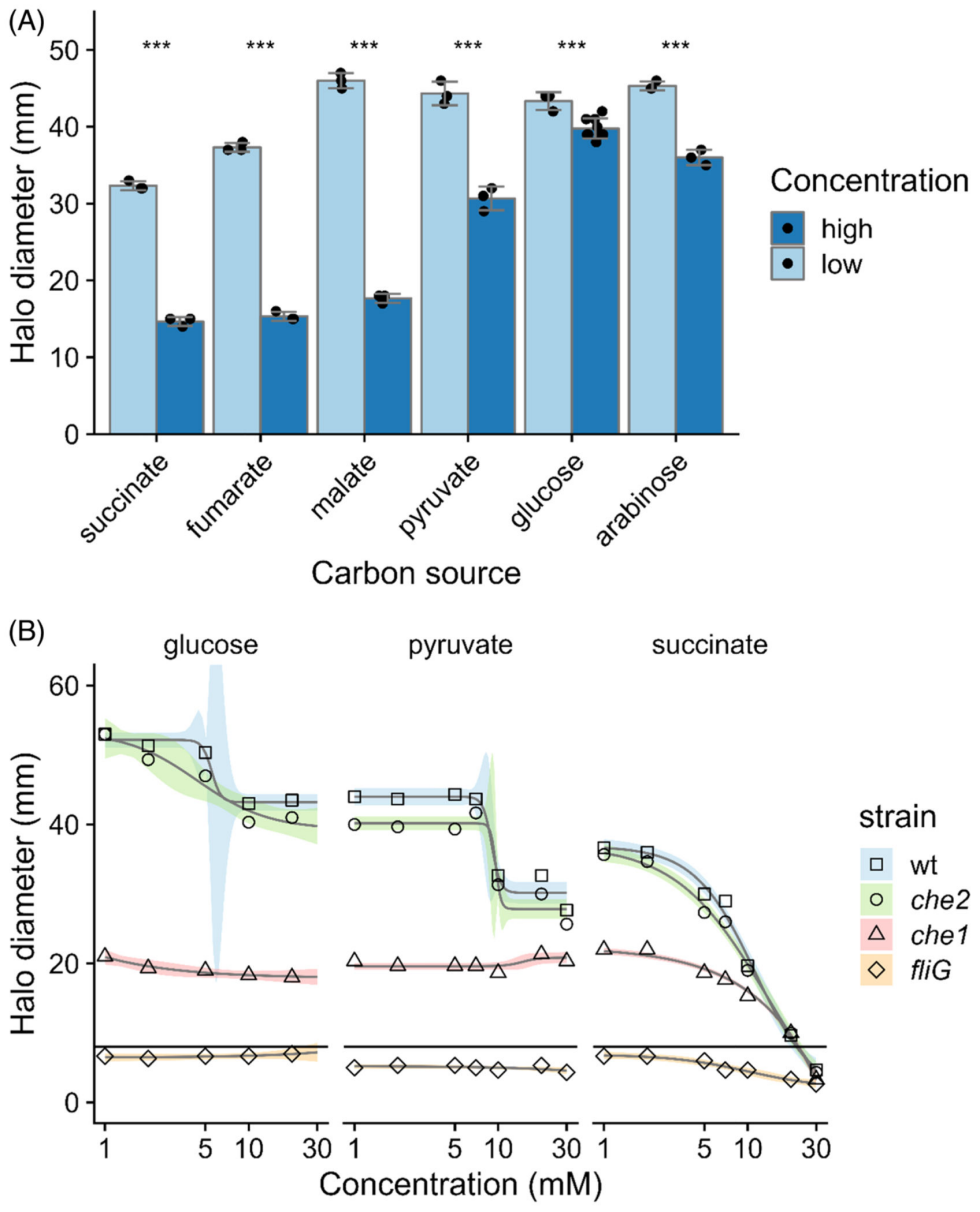
*R. leguminosarum* Rlv3841 swimming behaviour in different carbon sources. (A) Swimming halo diameters for Rlv3841 wildtype in semi-solid agar plates performed in UMS minimal media with 10 mM NH_4_Cl as N source and the corresponding C source tested as indicated, at low and high concentrations: 2 and 20 mM succinate, 2 and 20 mM fumarate, 2 and 20 mM malate, 3 and 30 mM pyruvate, 1 and 10 mM glucose, and 1 and 10 mM arabinose. Data were modelled with ANOVA and Dunnett’s post-hoc test comparing concentrations for each C source. Bars are mean ± SD, *N* = 3. ***p < 0.001. (B) Swimming halo diameters of wildtype (Rlv3841, squares) versus *che1* (LM100, triangles), *che2* (LM400, circles) and *fliG* (OPS2163, diamonds) on UMS minimal media with 10 mM NH_4_Cl and concentrations of glucose, pyruvate and succinate varying from 1 to 30 mM. Data were modelled with four-parameter Log-logistic or linear models for each strain and carbon source where appropriate. Shapes represent mean. Lines and ribbons are model predictions with 95% CI, *N* = 3. The horizontal line represents the estimated maximum non-motile diameter.

We first monitored wildtype swimming levels in semi-solid agar swimming plates with UMS minimal media supplemented with 10 mM NH_4_Cl and a wide range of carbon sources at low and high concentrations (10-fold difference). Rlv3841 displayed reduced halo diameters at higher carbon concentrations across all compounds, confirming that Rlv3841 swims more when starved (Wei & Bauer, 1998) (Figure 1A). The effects were particularly dramatic on the dicarboxylate intermediates of the TCA cycle (succinate, fumarate and malate), with precursors (pyruvate) and sugars (glucose and arabinose) showing minor effects. In addition, on TCA-cycle intermediates, the ring morphology was small with cloudy spots (see plates on succinate, Figure S5).

In Rlv3841, the two chemotaxis clusters Che1 and Che2 are implicated in the control of flagellar-based motility. To investigate these systems, we mutated a flagella stator protein (*motA*; OPS2107), a flagella rotor protein (*fliG*; OPS2163) and a flagella filament protein (*flaA*; OPS2234). Both *motA* and *fliG* mutants were confirmed to be non-motile with swimming assays on semi-solid UMS agar plates supplemented with 10 mM glucose and 10 mM NH_4_Cl (Figure S6A,C,H), while the *flaA* mutant displayed greatly reduced motility compared to the wildtype (Figure S6B,H). In addition, we used three previously generated mutants of the chemotaxis clusters: *che1* (LM100), *che2* (LM400) and the double mutant *che1*,*2* (LM300) (Miller et al., 2007). As already reported, the *che1* and *che1*,*2* mutants had much smaller swimming halos than the wildtype, whereas the *che2* mutant displayed only a minor reduction in halo diameter (Figure S6D–F,H), highlighting the relevance of the Che1 cluster in free-living motility.

Since the swimming plates only display overall responses to media conditions, to define the general motility parameters of Rlv3841 we tracked the swimming behaviour of individual cells in liquid culture capillary assays using video microscopy. Like the swimming plates, these assays were conducted under carbon starvation, where cells are highly motile, using 1 mM glucose (metabolized through glycolysis), 3 mM pyruvate (precursor of the TCA cycle) and 2 mM succinate (TCA-cycle intermediate). The tumble rates and speed values followed an inverted pattern, where the deletion of the *che1* cluster resulted in a significant reduction in tumbles combined with faster speeds, reinforcing the relevance of this chemotaxis cluster in free-living conditions (Table 1 and Figure S7). Overall, wildtype Rlv3841 swims at a rate of 44 ± 7, 38 ± 6 and 41 ± 7 μm per second in glucose, pyruvate and succinate, respectively, and has a tumble rate of 0.11, 0.14 and 0.08 per second in these same growth conditions.

To further analyse the dramatic reduction in chemotaxis and motility on high succinate, the swimming behaviour of wildtype compared to that of *che1, che2* and *fliG* mutants was assessed in semi-solid UMS agar plates supplemented with varying concentrations of glucose, pyruvate and succinate (Figure 1B). Rlv3841 and the *che2* mutant displayed modest reductions in the halo diameter with increasing carbon concentration, with a ~20% reduction in glucose and a ~30% reduction in pyruvate, whereas the *che1* mutant did not display any reductions. However, the results on succinate show a dramatic trend, with a 70% reduction in the swimming halo diameter for wildtype and the convergence of halo diameter formed by all tested strains at 30 mM succinate. Results with a similar trend were obtained for the *che1* mutant, indicating that the halting effect is not dependent on chemotaxis. In addition, all the strains tested displayed halos with the cloudy and spotty morphology described previously (Figure S5).

### Motility genes and the Che1 chemotaxis cluster are important in the competition for nodulation in pea by Rlv3841

**Figure 2:**
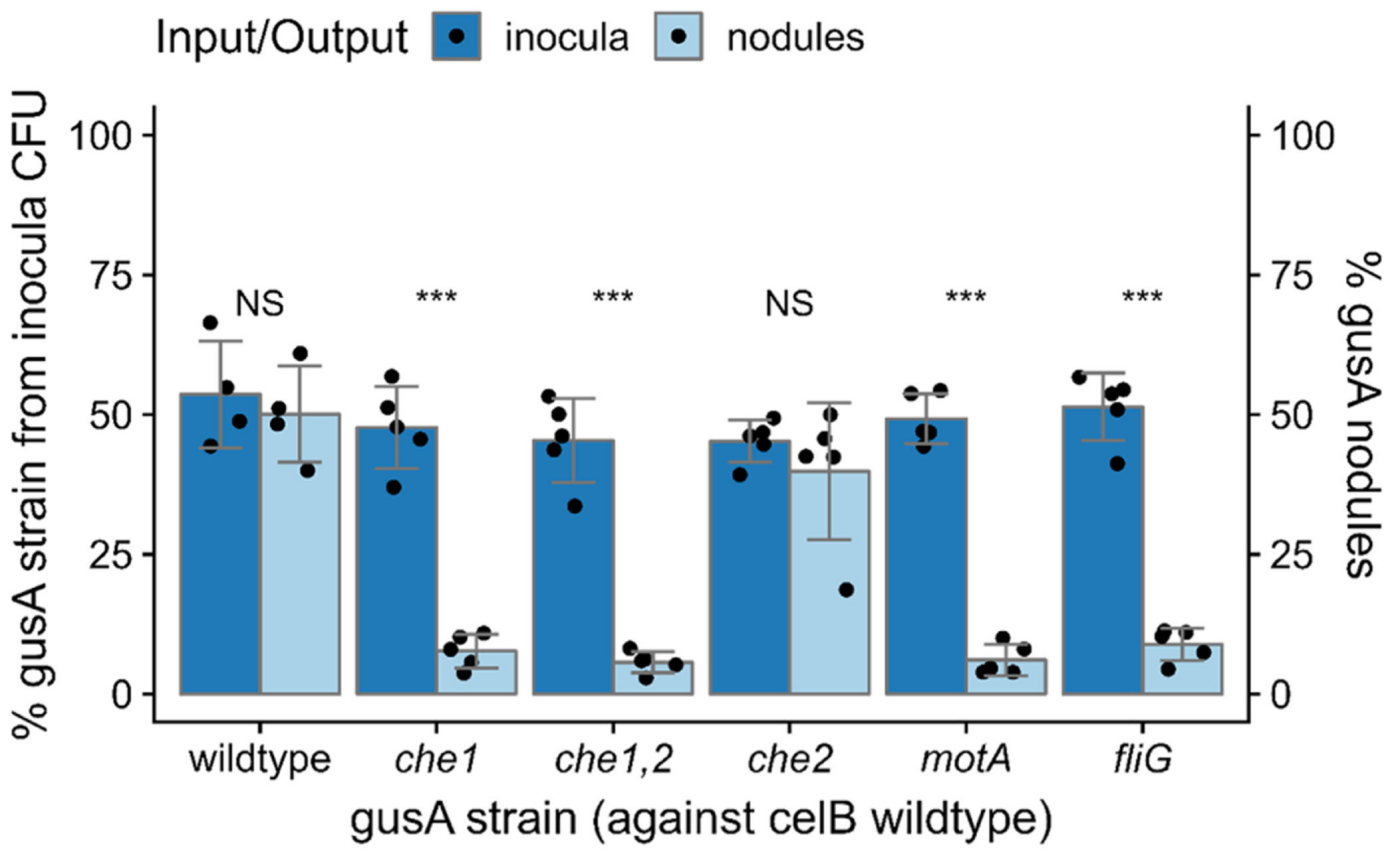
Competition assays on nodulation for chemotaxis and motility mutants. *gusA*-marked strains of wildtype (OPS1896), *che1* (OPS1871), *che1*,*2* (OPS1872), *che2* (OPS1873), *motA* (OPS2267) and *fliG* (OPS2447) mutants were tested against *celB*-marked wildtype (OPS2085). Dark bars indicate the CFUs in the initial inoculum counted after plating the 1:1 mixes (input) and pale bars indicate the number of *gusA* nodules (output) obtained from the total number of nodules counted for each plant root after 21 dpi and subsequently stained. Data were modelled with binomial GLM with the factors input/output, strain, and their interaction with random intercepts per plant. Input/output and the interaction factors were significant, *p* < 1e – 10, ANOVA. Dunnett’s post-hoc test found mean differences between input/output with NS (not significant), *p* > 0.05 and ***p < 0.001. Bars are mean ± SD, *N* = 5.

We measured the effects of chemotaxis and motility on competition for nodulation by inoculating pea plants with either the wildtype Rlv3841, *che1, che1*,*2, che2, motA, fliG* or *flaA* mutants, all tagged with *gusA*, against *celB*-tagged wildtype in a 1:1 ratio, proceeded by plant harvest after 21 dpi post inoculation (dpi) and sequential double staining with X-gluc and magenta-GlcA to enable nodule counting. We compared the strain ratio in the initial inoculant (input)—measured by plating the inoculant mixes and counting CFUs (colony forming units) - with the strain ratio reflected by the nodule counts (output). *che1, che1*,*2, motA* and *fliG* mutants had a significant reduction in competition for nodulation compared to wildtype by 85%, 89%, 88% and 84%, respectively (Figure 2), whereas the *che2* mutant did not show an effect. These results confirm previous studies where motility and the Che1 chemotaxis cluster were important for competitive nodulation (Miller et al., 2007).

### Che1 affects swimming to the roots, whereas Che2 affects nodule maturation

**Figure 3:**
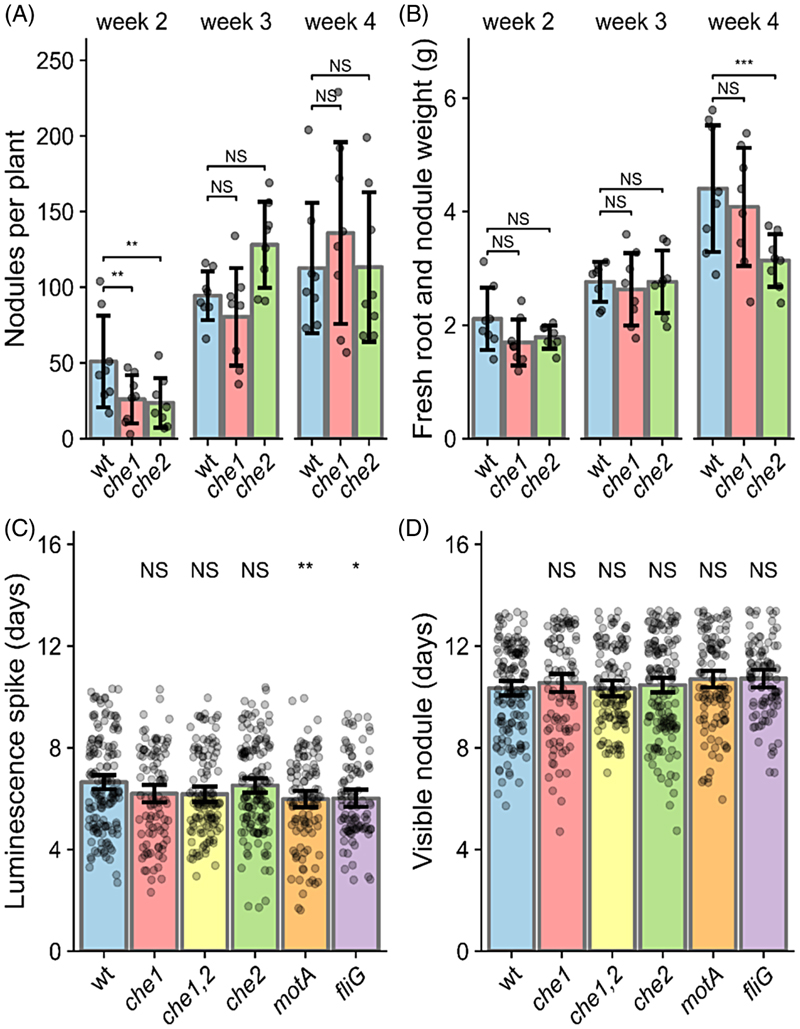
Nodulation development and dynamics. (A, B) Wildtype (OPS2085), *che1* (OPS2086) and *che2* (OPS2088) strains harbouring constitutive *celB* inoculated on pea plants in the sand: vermiculite pots and harvested after 2–4 weeks, when nodules were counted (A) and entire fresh root was weighed (B). Nodule counts were modelled with Poisson GLM ANOVA with Dunnett’s post-hoc test, *N* = 8. (C, D) Wildtype (LMB612), *che1* (OPS2139), *che1*,*2* (OPS2140), *che2* (OPS2141), *motA* (OPS2528) and *fliG* (OPS2526) strains harbouring a *pnodA::lux* promoter fusion inoculated on vetch plants grown on 1% agar FP media square plates. Plates were imaged daily to detect spikes of luminescence (C) and their corresponding first nodule appearance (D). Bars represent mean appearance time ± 95% CI, 5–8 independent growth plates each containing 5 plants, per strain. Data were modelled with ANOVA and Dunnett’s post-hoc test comparing wildtype to each strain. NS (not significant), *p* > 0.05, *p < 0.05, **p < 0.01, ***p < 0.001.

While the Rlv3841 Che1 cluster affects free-living cell motility and competitive nodulation, the Che2 cluster does not have an effect in either process. However, we previously showed by transposon insertion sequencing (INSeq) that the *che2* gene cluster seems to be involved in the later stages of bacterial movement and progression down infection threads (Wheatley et al., 2020), indicating that *che2* mutants might have slower nodule development. To further investigate the role of *che1* and *che2* chemotaxis clusters in nodule formation and nodulation dynamics, nodule count and fresh root weight (accounting for nodule mass) were measured over 4 weeks for pea plants individually inoculated at 10^5^ cells in sand: vermiculite pots with wildtype and these mutant strains, each marked with constitutively expressed *celB* to enable staining for nodule visualization. Following *celB*-staining, nodules were counted per plant after 2, 3 and 4 weeks post-inoculation harvests. Both the *che1* and *che2* mutants formed about 50% fewer nodules than the wildtype at week 2, although the numbers recovered by week 3 (Figure 3A). For the *che1* mutant, this is consistent with the already reported defective free-living motility (Figure S5 and S6) and competition of nodulation when co-inoculated with the wildtype (Figure 2), indicating that this competitive disadvantage occurs when swimming to the roots, prior to the formation of the root hair microcolony. The *che1* mutant does eventually recover nodule count after sufficient time for root hair colonization, showing no defect in nodule health as assessed by fresh root weight (Figure 3B). In contrast, the *che2* mutant was not outcompeted by the wild type in co-inoculation competition (Figure 2), but it also shows a reduced number of nodules in week 2 (Figure 3A). This suggests that *che2* slows later nodule development. For ease of discussion, we consider this later nodule development as maturation. This is also supported by the defect in nodule health by the fourth week, likely due to reduced nitrogen fixation by less mature nodules (Figure 3B).

To assess the role of chemotaxis and motility after root hair colonization, we did a time-course experiment to track the nodulation dynamics of the wildtype Rlv3841, *che1* and *che2* mutants. The strains contained plasmid pLMB712 (*pnodA::lux* promoter fusion), which produces spikes of luminescence when plant flavonoids induce rhizobial *nod* gene expression, resulting in Nod Factors (NFs) production. This is established when plant-microbe signalling is initiated, which happens before visible nodule formation (Pini et al., 2017). NF induce initial swelling of the root hair tip within minutes after NF application, whereas root hair deformation outgrowth leading to nodule formation generally initiates 50–60 min later (Esseling et al., 2003). To remove the motility requirements, we inoculated these strains directly along the root of *Vicia villosa* (hairy vetch) plants (a host of *R. leguminosarum*) grown on square plates with FP agar. Vetch plants allow a very precise *in vivo* identification of the local increase of *nod* gene-inducing flavonoids at the onset of the symbiosis. During this experiment, the time of nodule initiation events (visualized as localized spikes of luminescence, Figure 3C and S8A) and the initial appearance of each nodule (Figure 3D and S8B), were recorded. This experimental setup allowed us to confirm that there were no significant differences for the *che1* and *che2* mutants compared to wildtype for both processes, the signal initiation and nodule detection. However, significant time reductions were found for the motility mutants *motA* and *fliG* compared to wildtype, with *nodA* spikes recorded on average half a day earlier (Figure 3C), although this difference did not impact nodule appearance, where *motA* and *fliG* mutants showed no significant differences (Figure 3D). These results indicate that loss of flagellar motility or the main chemotaxis systems does not impede nodule initiation when rhizobia do not need to swim to get into the roots. Therefore, since rhizobia must have colonized plant roots when *nodA* induction occurs, the Che1 defect is likely to occur during movement to root hair tips and that of Che2 during infection thread formation and/or nodule maturation.

### The Che2 chemotaxis system influences bacteroid differentiation

**Figure 4:**
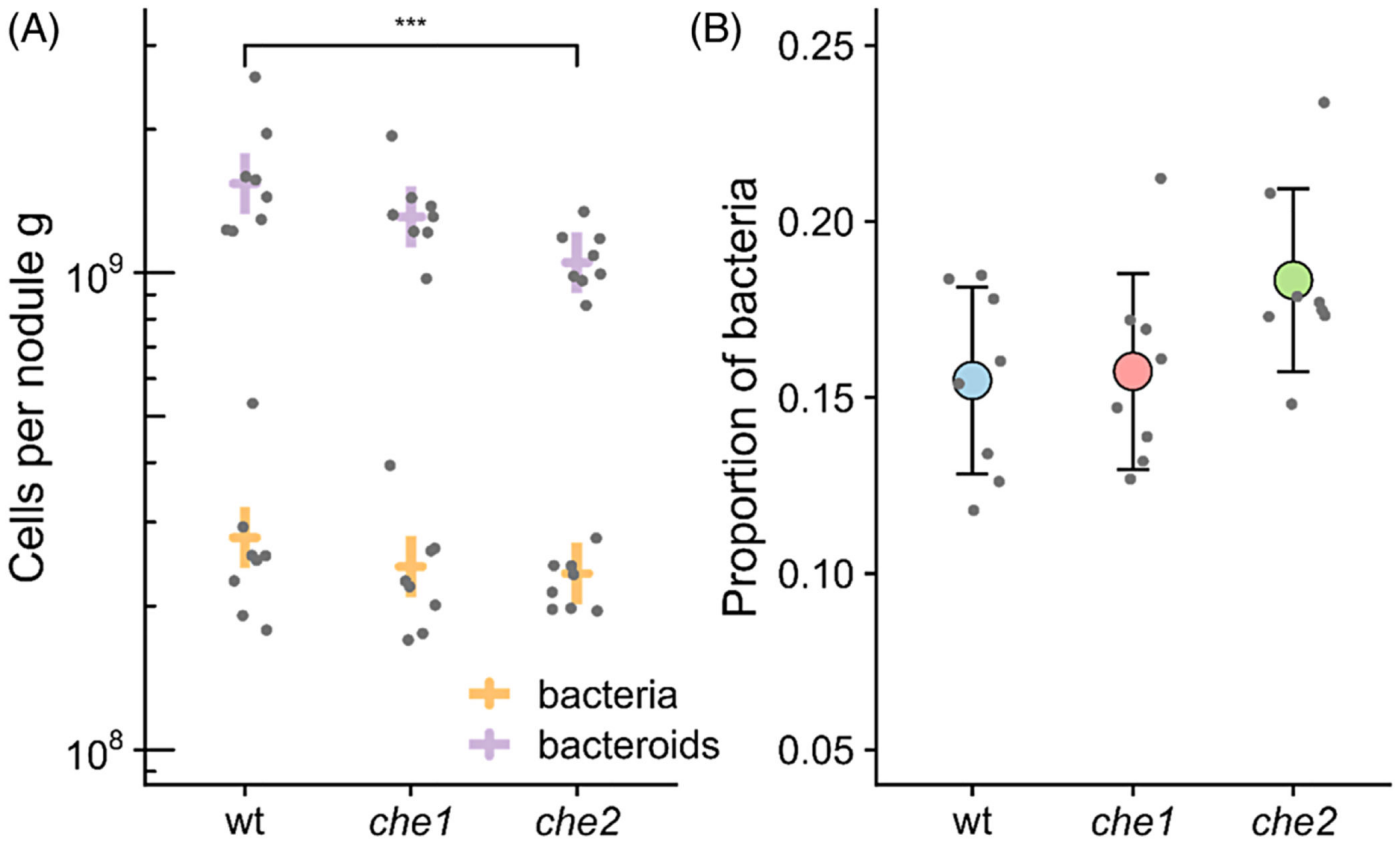
Undifferentiated bacteria versus bacteroid counts per nodule. The strains tested were sfGFP marked Rlv3841 wildtype (OPS1953), *che1* (OPS1847) and *che2* (OPS1859). (A) Cells per nodule gram obtained from 10 nodules per each plant sampled, indicating in yellow the counts for undifferentiated bacteria and, in purple, the bacteroid counts. Data were modelled with Poisson GLM with the factors strain and cell type (bacteria or bacteroids) with random intercepts per plant and offset by volume and nodule mass. The single terms were all found to be significant, *p* < 1e – 10, ANOVA. Dunnett’s post-hoc test found mean differences between strains with NS *p* > 0.05, ***p < 0.001. Lines are model prediction ± 95% CI with samples as points, *N* = 8. (B) Proportion of undifferentiated bacteria among cells inside the nodules formed by the above strains. Circles represent the mean ± SD with individual plant samples shown as points, *N* = 8.

Impacts on nodule development and maturation are likely to result in altered bacteroid differentiation. We thus individually inoculated pea plants with wildtype and, *che1* and *che2* mutant strains, each marked with constitutively expressed sfGFP, and counted the cell populations of undifferentiated bacteria vs. bacteroids via flow cytometry in mature individual nodules formed in pea plants harvested at 21 dpi. We distinguished bacteria and bacteroids by size and granularity, measured as forward and side scatter, respectively. While there were no significant differences in the numbers of bacteria per gram of nodule, we found a significant reduction in the *che2* mutant bacteroid count compared to the wild type (Figure 4A). This also resulted in a higher proportion of bacteria relative to bacteroids in the nodules formed by the *che2* mutant (Figure 4B). This apparent defect in bacteroid development is consistent with Che2 retarding nodule maturation.

## Regulation of motility by PTS^Ntr^ in Rlv3841

**Figure 5:**
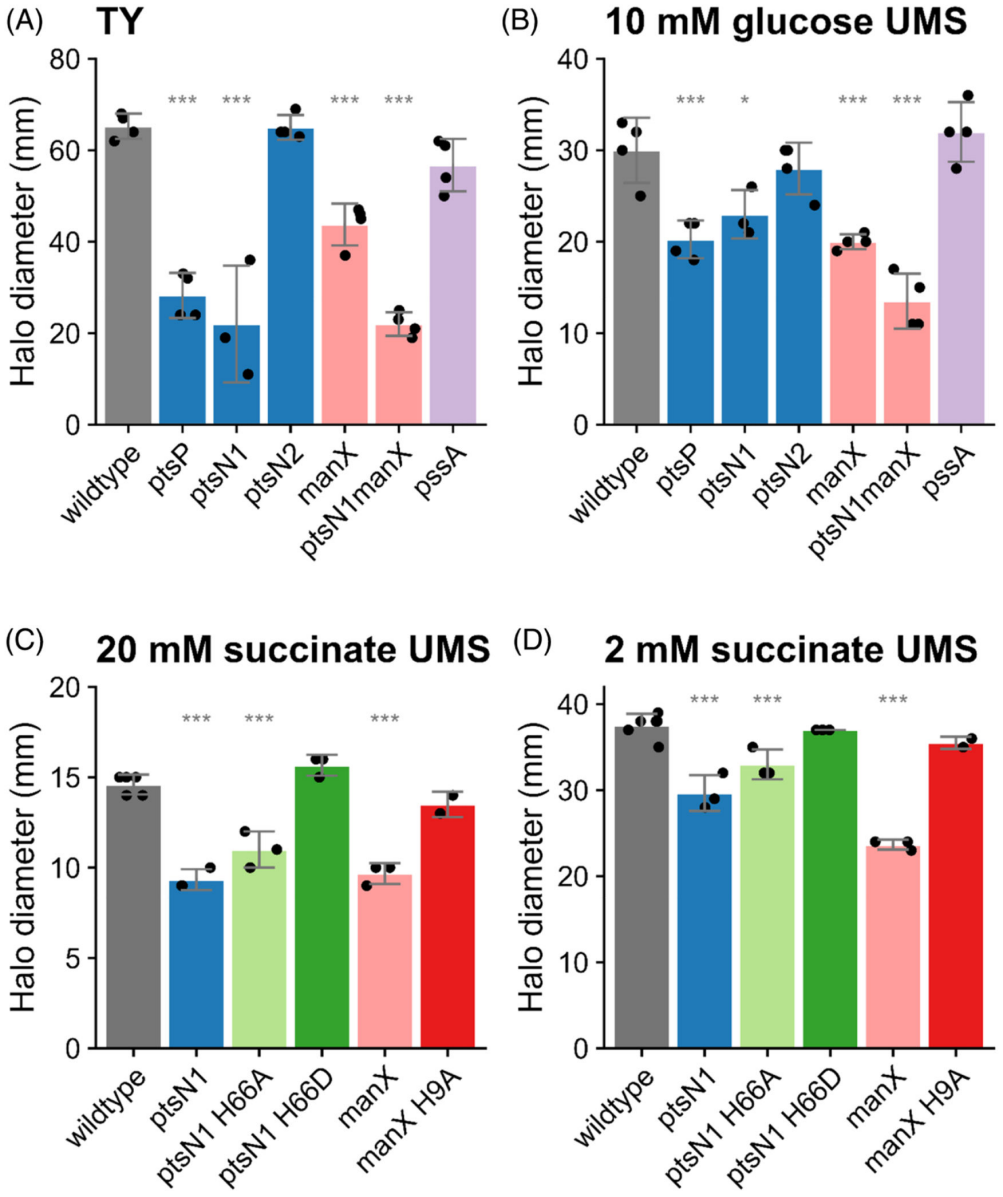
PTS regulates motility under different nutrient conditions. Swimming halo diameters in semi-solid agar plates for wildtype (grey), *pstP* mutant (PstP107, blue), *ptsN1* mutant (LMB271, blue), *pstN2* mutant (RU4391, blue), *ptsN1* H66A (OPS1102, pale green) *ptsN1* H66D (OPS1104, dark green), *manX* mutant (LMB692, pink), *ptsN1 manX* double mutant (OPS0374, pink), *pssA* mutant (LMB310, purple) and *manX* H9A (OPS1012, red) on (A) TY media and UMS media with 10 mM NH_4_Cl as N source and (B) 10 mM glucose; (C) 20 mM succinate or (D) 2 mM succinate as C sources. Data were modelled with ANOVA and Dunnett’s post-hoc test comparing strains to wild type. Bars are mean ± SD, *N* = 3–4. No star annotation indicates no significant difference *p* > 0.05, *p < 0.05, **p < 0.01, ***p < 0.001.

Since the relationship between motility and metabolism remained unclear, we investigated PTS^Ntr^ as a candidate for metabolism-based control of motility. We first conducted swimming assays in soft-agar plates for all the PTS^Ntr^ mutants in both rich (TY) and minimal (UMS) media supplemented with 10 mM NH_4_Cl and 10 mM glucose. In both conditions, we observed a clear reduction in the halo size for all mutants, except for *ptsN*_*2*_—a second copy of *ptsN* with an unknown role (Figure 5A,B). Indeed, the double mutant *ptsN*_*1*_*manX* showed the strongest effect in either rich or minimal media (TY and UMS with 10 mM glucose) and was independent of the differences in exopolysaccharide (EPS) production reported previously, as the *pssA* mutant defective in EPS production was fully motile. These results suggest that there is a regulatory link between PTS^Ntr^ and motility in Rlv3841, so we examined the effect of the downstream proteins ManX and PtsN in more detail. We screened the swimming behaviour of these mutants in minimal-media soft-agar plates under high and low succinate concentrations, since these were the conditions with the most dramatic differences in swimming behaviour. As expected, the *manX* mutant displayed significantly smaller halos than wildtype and this phenotype was complemented by the non-phosphorylatable version of ManX (H9A, Man-X*Ala) (Figure 5C,D). However, the *manX* mutant was previously reported as growth defective in several carbon and nitrogen sources, including 10 mM glucose and 20 mM succinate (Sánchez-Cañizares et al., 2020). Since the *ptsN*_1_ mutant does not show a growth defect and displayed significantly smaller halos than wildtype that were complemented by the phosphomimic version (H66D; PtsN*P) (Figure 5C,D), our results point to a connection between the PTS^Ntr^ system and motility in rhizobia that will need to be explored further.

## Discussion

The number of MCPs per rhizobial genome varies greatly, with less than 10 in *Sinorhizobium* and *Ensifer* spp. to over 30 in *R. leguminosarum* or *Bradyrhizobium japonicum* isolates (Scharf et al., 2016), although only a few of them have assigned functions (Compton & Scharf, 2021; Salar et al., 2023). Genome analysis revealed that Rlv3841 has 27 predicted chemoreceptors, with a wide variety of sensory domains and phylogenetic diversity. Together, they have periplasmic sensors predicted to sense amino acids, peptides, carbohydrates, carboxylates and organic acids, and cytoplasmic sensors predicted to sense oxygen, redox levels or light. Characterization of the domains of the chemoreceptors revealed two distinct groups: those encoding proteins with longer signalling domains associated with the *che2* cluster (*mcrA, mcrB* and *mcrC*) and those with smaller signalling domains. The matching smaller signalling domains of most Rlv3841 chemoreceptors indicates that they may form a common chemosensory array that likely signals through the Che1 cluster, which in turn, might be what drives the dominance of the Che1 cluster (Briegel et al., 2009; Yang & Briegel, 2020). Some of the receptors were found to not have transmembrane domains, indicating they might act as internal sensors that are likely responding to the metabolic state of the cell, reacting with a form of energy taxis—driving the bacterium towards regions with maximal energy production (Alexandre & Zhulin, 2001; Taylor et al., 1999). Future work is needed to characterize these chemoreceptors and elucidate the signals they sense.

We describe the swimming behaviour of Rlv3841 wildtype, chemotaxis and flagellar mutants in varying concentrations of carbon sources, clearly showing that instead of shutting off motility to conserve energy and substrates, Rlv3841 swims most when starved. This reflects how, in natural environments, continued motility and chemotaxis may offer a bacterial population its best chance to find new nutrient supplies as nutrient availability approaches zero (Wei & Bauer, 1998). In this work, we also discovered a novel motility-modulating system in the symbiont Rlv3841 that responds to high concentrations of TCA-cycle intermediates to halt motility. This dicarboxylate halting effect was independent of both chemotaxis systems, also occurring in the *che1* and *che2* mutants. One possible mechanism mediating this effect would be the flagellar transcriptional control through a TCA-cycle intermediate sensing regulator, like ActSR (Elsen et al., 2004; Fenner et al., 2004; Priefer et al., 2001; Tiwari et al., 1996).

We also optimized a cell tracking pipeline in free-living conditions to determine the motility parameters of Rlv3841 in various media conditions, including the swimming speed (38–41 μm/s) and number of tumbles (0.08–0.14 /s). These agree broadly with Rlv3841 swimming speeds of 38.3 ± 6.7 μm per second and tumbles at 0.34 ± 0.01 per second found previously on mannitol media (Miller et al., 2007). Swimming speeds can vary greatly between bacterial species: *E. coli* swims at an average rate of 25–32 μm per second (Ahmed & Stocker, 2008; Mitchell & Kogure, 2006), *Pseudomonas aeruginosa* at 23.9 μm per second (Khong et al., 2021), whereas *Bdellovibrio bacteriovorus* can reach 160 μm per second (Lambert et al., 2006). Other rhizobia have comparable swimming speed to Rlv3841, *Rhizobium lupini* swims at 33–43 μm per second (Scharf, 2002) and *S. meliloti* at 23–31 μm per second (Sourjik & Schmitt, 1996).

Chemotaxis and motility are known components of rhizobial competition in the early infection stages leading to nodule occupancy, with mutants greatly disadvantaged in nodulation competition with wildtype (Bauer & Caetano-Anollés, 1990; Miller et al., 2007; Wadisirisuk et al., 1989; Yost et al., 1998). Our competition experiments performed in Rlv3841 confirmed a significant reduction in the number of nodules formed by *che1, che1*,*2, motA* and *fliG* mutants when tested against the wildtype strain (Figure 2), in agreement with previously reported nodulation competition and INSeq data (Miller et al., 2007; Wheatley et al., 2020). In addition, we found that when inoculated individually, both the *che1* and *che2* mutants formed fewer nodules than the wildtype in the second week (Figure 3A). To further investigate the cause of the competition defect, our next experiment investigated nodulation dynamics, measuring early infection start time with a flavonoid biosensor and the first nodule appearance (Pini et al., 2017). Nodule development timings were recorded for when the luminescence spike was first observed (Figure 3C), reflecting the initial signalling, and when the nodule first appeared in that root spot (Figure 3D). These developmental stages were not significantly different for the *che1* or *che2* mutants.

In this work we found that the *che2* mutant was not significantly different from the wildtype in either nodule competitiveness or timing for nodule appearance (Figures 2 and 3D), agreeing with previous studies (Miller et al., 2007). This indicates that the *che2* cluster does not have a significant effect on swimming to root or on surface movement to root hairs (e.g., swarming or twitching). However, during indeterminate nodule maturation as in pea and vetch plants, bacteroids are continually formed through terminal differentiation, forming large, branched and non-viable cells optimized for nitrogen-fixation (Mergaert et al., 2006; Poole et al., 2018). Thus, we examined the impact of the *che2* deletion on bacteroid differentiation by comparing the composition of wildtype nodules to those of the *che2* mutant, counting the undifferentiated bacteria and bacteroids extracted from mature pea nodules. We found that although there was no significant difference between counts of undifferentiated bacteria from wildtype and *che2* mutant nodules, there was a 30% reduction of bacteroids (Figure 4A,B). Since the *che2* mutant forms nodules with bacteroids showing a maturation defect compared to wildtype, mutants with insertions inside the cluster are expected to show a nodule developmental defect, as reflected by the reduction in fresh root and nodule weight in the *che2* mutant on week 4 (Figure 3B).

**Figure 6:**
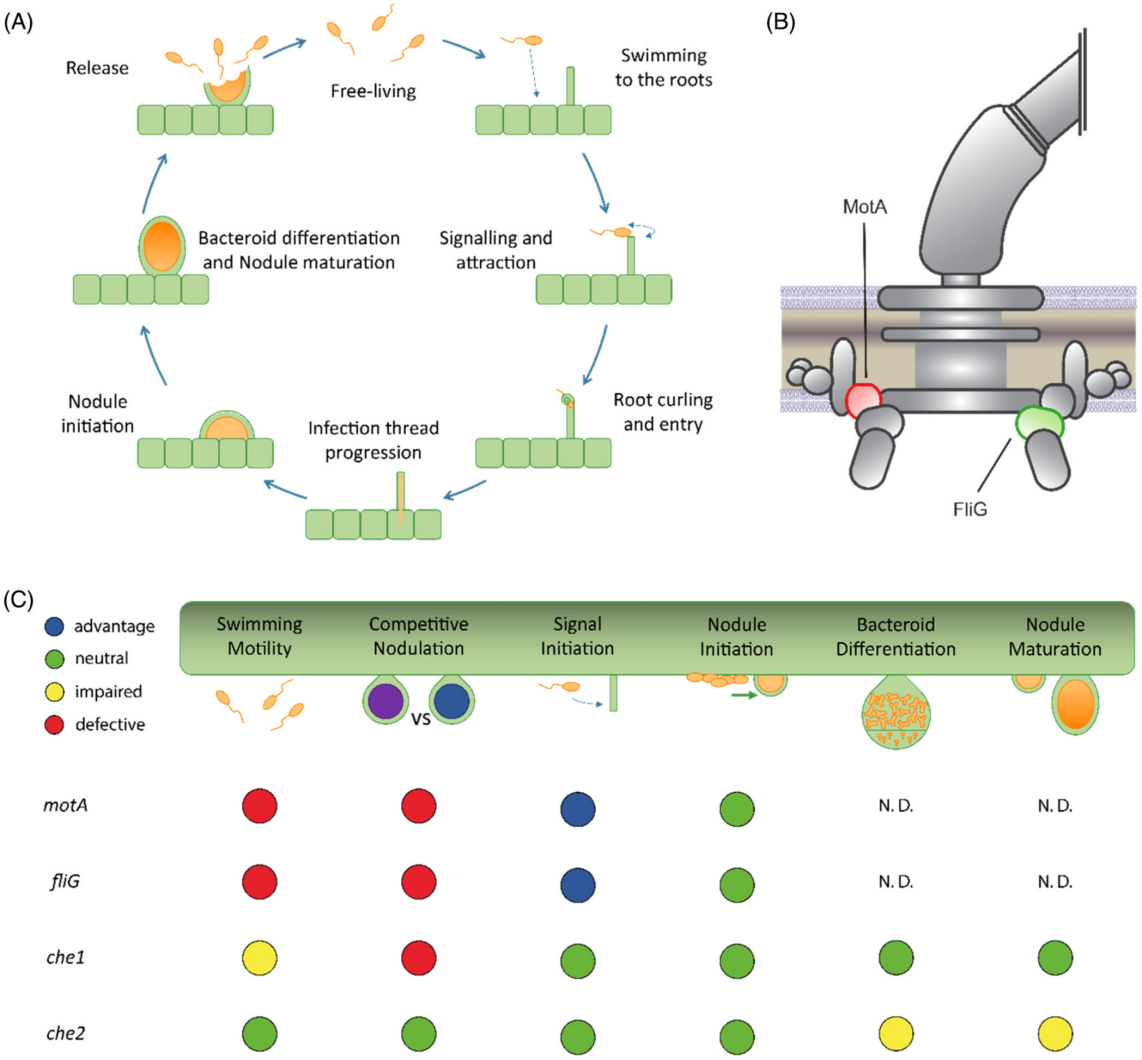
Motility and chemotaxis effects across the *Rhizobium*-legume symbiosis. (A) Rhizobia are initially living in the soil as free-living cells (free-living), they then move toward the plant root surface through motility and chemotaxis (swimming to the roots), where they engage in a molecular dialogue with the plant and colonize the root (signalling and attraction). If successful, this recognition between the two partners enables their entrance into the root hair (root curling and entry). The plant responds to bacterial entry by forming infection threads that grow and ramify into plant cortical cells. Bacteria move down the infection threads and are eventually endocytosed by plant cells where they start to form a nodule (nodule initiation) and differentiate into nitrogen-fixing bacteroids (nodule maturation). Eventually, the nodule undergoes senescence, releasing the undifferentiated nodule bacteria back into the soil (release). (B) Model of Rlv3841 flagella highlighting the position of the motor stator MotA and the motor rotor FliG proteins used in this study. (C) Model showing the effects of motility and chemotaxis systems across key stages in the symbiotic process summarizing the results obtained in this work from deleting two genes encoding flagellar proteins (*motA* and *fliG*) and both chemotaxis clusters (*che1* and *che2*) in Rlv3841. Blue circles represent an advantage, green circles represent no effect of the mutation, yellow circles—a minor effect, and red circles—a major effect. N.D. not determined.

We therefore speculate that the main chemotaxis system Che1 is needed for swimming to the roots, as reflected by the defective free-living swimming and competitive nodulation, whereas Che2 is needed at later stages for nodule maturation, suggested by the lower number of differentiated bacteroids, as proposed in the model presented in Figure 6. *S. meliloti* chemotaxis and motility mutants were also found to nodulate alfalfa at a reduced rate with delayed nodule emergence (Bauer & Caetano-Anollés, 1990; Caetano-Anollés et al., 1988). Furthermore, an *Azorhizobium caulinodans* chemoreceptor mutant was found to have reduced competition for nodulation with *Sesbania rostrata* (Liu et al., 2017). Interestingly, the reduced efficiency of the *S. meliloti* and *A. caulinodans* mutants could not be completely attributed to improved root colonization, leading to speculation that motility and chemotaxis are important for further stages of symbiosis, such as movement to appropriate sites of infection (Caetano-Anollés et al., 1988). The Che2 chemotaxis system may be involved in bacteroid differentiation or perhaps in the movement down infection threads. As mentioned above, growth and cell division are the driving forces extending infection threads but there is evidence that some other form of motility is involved (Brewin, 2004; Fournier et al., 2008; Gage, 2004). Another unknown is if the Che2 system acts via flagella, pili or another, unknown system, to control swimming or surface motility such as swarming or twitching. Flagella is the most likely effector since the Che2 system has a known connection to swimming, with *che2* deletion mutants displaying reduced halo diameters and increased tumble rate, and CheB2 being known to methylate Che1-associated chemoreceptors (Miller et al., 2007). Although transcription of *che* and *fla* genes is indeed downregulated in nodules, this is likely to reflect the dominance of sessile bacteroids (Karunakaran et al., 2010). Future experiments need to determine whether the flagella are still present in infection threads and if the *che2* mutant effects are through their modulation of flagella.

To understand the global role of PTS^Ntr^ as a sensor of nutrient availability and determine if it is involved in the regulation of motility in Rlv3841, we analysed the swimming behaviour of a panel of PTS^Ntr^ mutants. All Rlv3841 PTS^Ntr^ mutants were defective in motility on soft-agar plates. The link between PTS and motility was first identified in *Vibrio fischeri* (Visick et al., 2007), where they showed that the carbohydrate PTS controls motility. In *V. vulnificus* dephosphorylated EIIA^Glc^ inhibits flagellar synthesis and swimming motility (Park et al., 2016). Similarly, PTS^Ntr^ also controls motility in *Caulobacter crescentus* and *E. coli* a *ptsP* mutant has reduced motility on swarm agar (Sanselicio & Viollier, 2015). PtsN interacts with proteins related to chemotaxis and flagellar assembly (Gravina et al., 2021), respectively. In Rlv3841, although both the carbohydrate (ManX) and PTS^Ntr^ (PtsN) branches appeared to alter motility, the *manX* mutant reduced swimming behaviour was complemented by a non-phosphorylatable version (H9A; ManX*Ala), whereas the reduced swimming by the *ptsN*_*1*_ mutant was complemented by the phospho-mimic version (H66D; PtsN*Asp). Since both proteins tend to be phosphorylated under nitrogen limitation conditions (Sánchez-Cañizares et al., 2020), this indicates a coupled control of motility that depends on phosphorylation, so that under nitrogen limitation, PtsN~P (phosphorylated PtsN) might stimulate motility, whereas unphosphorylated ManX stimulates motility under nitrogen-rich conditions. However, the growth defect of the *manX* mutant (Sánchez-Cañizares et al., 2020) needs to be considered. Since the *ptsN*_*1*_ mutant does not show a growth defect, we speculate that the PTS^Ntr^ control of motility exerted by PtsN_1_~P might indeed be mediated by the two-component regulatory system ChvGI, known to prevent motility gene activation in *Agrobacterium tumefaciens* (Alakavuklar et al., 2021; Heckel et al., 2014), and a direct target of phosphorylated PtsN_1_ (Sánchez-Cañizares et al., 2020), good evidence for a connection between the PTS^Ntr^ system and motility. Future work needs to be done to elucidate this regulatory mechanism.

Taken together, the data presented here illustrate the motility and chemotaxis properties of Rlv3841 as a model *α*-proteobacterium in plant–microbe interactions. We determine the role of motility and chemotaxis at various stages of the symbiotic process, particularly where competition takes place, and examine the complexities of the control of motility, showing the link to PTS^Ntr^ regulation and between central metabolic pathways and cell motility. Finally, we show that the Che1 cluster, in coordination with flagella, has a role in free-living motility and nodule competition, whereas the Che2 cluster may influence bacteroid development and nodule maturation. Motility and chemotaxis play important roles throughout the *Rhizobium*-legume symbiosis cycle and as such are critical to our understanding of its initiation, development and regulation.

## Supplementary Material

Supplementary file

## Figures and Tables

**Table 1:** *Rhizobium leguminosarum* swimming statistics.

	Average speed (μm/s)				Tumble rate (/s)		
	Glucose	Pyruvate	Succinate		Glucose	Pyruvate	Succinate
wildtype	44 ±7	38 ±6	41 ± 7		0.11 ±0.23	0.14 ±0.24	0.08 ±0.19
*chel*	54 ±8*	55 ±8*	50 ± 8*		0.08 ±0.19*	0.09 ±0.19*	0.05 ±0.14*
*che1,2*	58 ± 10*	57 ±9*	55 ± 8*		0.08 ±0.19*	0.12 ±0.22*	0.07 ±0.16*
*che2*	50 ±8*	45 ±7*	44 ± 7*		0.11 ±0.23	0.14 ±0.25	0.1 ±0.21*

*Note*: Average swimming speed and tumble rates calculated from individual tracks of *R. leguminosarum* strains in UMS minimal media supplemented with 1 mM glucose, 3 mM pyruvate and 2 mM succinate. Ten thousand to forty thousand tracks per group collated from three independent experiments. ANOVA with Dunnett’s post-hoc test comparing strains to wildtype.

* *p* < 0.001.

## Data Availability

The raw data associated with flow cytometry experiments have been uploaded to the FlowRepository public database: (http://flowrepository.org/experiments/6986) and all the pipelines developed for image analysis are publicly available on GitHub: https://github.com/AroneyS
