# Supplementary material for: The motility and chemosensory systems of Rhizobium leguminosarum, their role in symbiosis, and link to PTSNtr regulation
Source: Environ Microbiol. Author manuscript; Available in PMC 2025 Jul 22. (PMC7617929; doi:10.1111/1462-2920.16570)
Supplement: Supplementary file [file EMS206647-supplement-Supplementary_file.pdf]

Supplementary information

The motility and chemosensory systems of *Rhizobium leguminosarum*, their role in symbiosis and link to PTS^{Ntr} regulation

Samuel T. N. Aroney,[1] Celia Kessler,[1] Francesco Pini,[1] Philip S. Poole[1] and Carmen Sánchez-Cañizares[1*]

[1]Department of Biology, University of Oxford, South Parks Road, Oxford, OX1 3RB, UK

*Corresponding author: carmen.sanchez-canizares@biology.ox.ac.uk

Supplementary Information - Materials and Methods

Generation of stable mutants and tagged strains

motA, fliG and *flaA* mutants were isolated using the pK18mobSacB vector for stable double recombinants. Primers were designed to amplify regions of approximately 1,000 bp precisely flanking the *motA*, *fliG* and *flaA* gene regions (see Supplementary Table S4). pK18mobSacB vector was digested with SmaI (Thermo Fischer) and the resulting PCR inserts amplified using Q5 polymerase (New England Biolabs) were cloned with NEBuilder Hi-Fidelity (New England Biolabs), generating the plasmids pOPS1412, pOPS1422 and pOPS1423, respectively. The plasmids were transformed into *E. coli* DH5α and confirmed by PCR mapping with M13F+M13R primers and subsequent Sanger sequencing (Eurofins MWG Operon). Confirmed plasmids were then transformed into *E. coli* ST18, grown with a supplement of 50 $\mu g \cdot mL^{-1}$ 5-aminolevulinic acid (ALA). These were then conjugated into the recipient Rlv3841 by biparental mating onto selective kanamycin TY without supplemented ALA (Thoma and Schobert, 2009). Positive colonies were then inoculated into liquid non-selective TY media and spread onto 10% (v/v) sucrose TY media for *sacB* counter-selection (Hmelo *et al.*, 2015). Primers used to map deletion mutants were oxp3919+oxp3920 (*motA*, OPS2107), oxp3937+oxp3938 (*fliG*, OPS2163) and oxp3925+oxp3926 (*flaA*, OPS2234).

To assess the importance of the motility and chemotaxis systems at the first stages in symbiosis, we used the pLMB712 plasmid, which contains the *lux* cluster under the promoter of the Rlv3841 *nodABC* operon (pRL100185-7). This promoter is activated by flavonoids produced by pea plant roots at infection zones (Pini *et al.*, 2017) and we conjugated it into the *che1* mutant LM100 (named OPS2139) and the *che2* mutant LM400 (OPS2141) by

triparental mating using the helper plasmid pRK2013. Nitrofurantoin (20 $\mu g \cdot mL^{-1}$) was used to select against *E. coli*.

Strains were fluorescently marked with mini-Tn7 integration into the chromosome after the *glmS* gene by tri-parental matings to additionally mobilise the transposase plasmid pTNS3 (Choi and Schweizer, 2006). Each strain was marked with sfGFP (Pédelacq *et al.*, 2006) under a constitutive J23104 promoter using plasmids pOPS0858 (Guha *et al.*, 2022). Insertions were confirmed with by PCR with primers oxp2327 and oxp3060.

To carry out nodule staining, two marker genes were used: *gusA* (encoding a *β*-glucuronidase that turns magenta with Magenta-GlcA) and *celB* (thermostable *β*-galactosidase that turns blue with X-Gal) (Sánchez-Cañizares and Palacios, 2013). These markers were integrated with Tn7 as above, under the constitutive J23104 promoter, using plasmids pOPS0857 and pOPS1376, for *gusA* and *celB*, respectively. These plasmids were integrated into Rlv3841 wildtype, the *che1* mutant LM100, the *che2* mutant LM400 and the *motA* mutant OPS2106, resulting in the strains OPS1896, OPS1871, OPS1873 and OPS2267. Additionally, *celB* was also integrated on the Rlv3841 wildtype (OPS2085) as an experimental control.

Supplementary Information - Tables

Table S1. The chemoreceptors identified in the genome of Rlv3841. aa: amino acids.

Gene number	Gene name	Sensory domain	Sensor type	Predicted sensing
RL0426	*mcpX*	dCache1	Periplasm	Quaternary ammonium
RL0429	*hemAT*	Protoglobin	Cytoplasm	Oxygen
RL0455	*mcpT*	Unknown	Periplasm	Mono-, di- and tri-carboxylates
RL0564	*mcpE*	Unknown	Periplasm	-
RL0685	*icpA*	Protoglobin	Cytoplasm	Unknown
RL0757	*mcpZ*	Unknown	Periplasm	-
RL0758	*mcpI*	Cache3-Cache2	Periplasm	aa and small carbohydrates
RL0949	*mcpJ*	sCache2	Periplasm	aa and small carbohydrates
RL0972	*mcpK*	CHASE3	Periplasm	Small peptides
RL1065	*mcpL*	4HB	Periplasm	Unknown
RL1318	*mcpM*	dCache1	Periplasm	aa and small carbohydrates
RL1386		N/A	N/A	N/A
RL1447		N/A	N/A	N/A
RL2683	*mcpD*	Unknown	Periplasm	-
RL2931	*mcpN*	sCache2	Periplasm	Short-chain carboxylates
RL3985	*mcpO*	dCache1	Periplasm	aa and small carbohydrates
RL4031	*mcrA*	4HB	Periplasm	Unknown
RL4032	*mcrB*	4HB	Periplasm	Unknown
RL4033	*mcrC*	4HB	Periplasm	Unknown
RL4277	*mcpP*	Unknown	Periplasm	-
RL4387	*mcpQ*	PAS	Cytoplasm	Oxygen, redox, light
pRL80031	*mcpS*	sCache2	Periplasm	aa and small carbohydrates
pRL100403	*mcpG*	4HB	Periplasm	Unknown
pRL120056	*mcpR*	Unknown	Periplasm	-
pRL120068	*mcpY2*	PAS	Cytoplasm	Oxygen, redox, light
pRL120312	*mcpC*	4HB	Periplasm	Unknown
pRL120683	*mcpB*	HBM	Periplasm	Organic acids

Table S2. Strains

Name	Description	Reference
DH5α	*E. coli* supE44, hsdR17, recA1 thi-1, ΔlacU169(ϕ80*lacZ*ΔM15) endA1 gyrA96 relA1	Hanahan (1983)
ST18	*E. coli* S17 $\lambda pir \Delta hemA$	Thoma & Schobert (2009)
Rlv3841	*R. leguminosarum* bv. *viciae*; Str^R derivative of strain 300	Johnston & Beringer (1975)
RU1235	Rlv3841 carrying pRU583 (Rlv3841 phoA::GFP)	Allaway et al. (2001)
Sme2011	*Sinorhizobium meliloti* 2011 *str3*; Str^R derivative of strain SU47	Boucher et al. (1977)
LM100	Rlv3841 *Δche1*	Miller et al. (2007)
LM300	Rlv3841 *Δche1,2*	Miller et al. (2007)
LM400	Rlv3841 *Δche2cheBII*	Miller et al. (2007)
PtsP107	Rlv3841 Tn5::*ptsP*	Prell et al. (2012)
LMB271	Rlv3841 *ptsN1*::ΩSpec	Prell et al. (2012)
RU4391	Rlv3841 *ptsN2*::ΩTet	Prell et al. (2012)
LMB310	Rlv3841 *pssA1* Tn5::spec transductant	Prell et al. (2012)
LMB692	Rlv3841 *manX* in-frame deletion	Sánchez-Cañizares et al. (2020)
LMB612	Rlv3841 carrying pLMB712 (Rlv3841 pnodA::lux)	Pini et al. (2017)

AA047	Rlv3841 *ptsN2* (markerless mutation) in *ptsN1*::ΩSpec background (Rlv3841 *ptsN1/N2* double mutant)	Sánchez-Cañizares et al. (2020)
OPS0374	Transduction of *ptsN1*::ΩSpec into LMB692 (Rlv3841 *ptsN1/manX* double mutant)	Sánchez-Cañizares et al. (2020)
OPS1012	LMB601 with pOPS0375 used to replace *manX*::ΩSpec with *manX* H9A (Rlv3841 with non-phosphorylatable ManX)	Sánchez-Cañizares et al. (2020)
OPS1102	AA047 with pOPS0373 used to replace *ptsN1*::ΩSpec with *ptsN1* H66A (Rlv3841 ptsN2 mutant with non-phosphorylatable PtsN1)	Sánchez-Cañizares et al. (2020)
OPS1104	AA047 with pOPS0374 used to replace *ptsN1*::ΩSpec with *ptsN1* H66D (Rlv3841 *ptsN2* mutant with PtsN1 phosphomimic)	Sánchez-Cañizares et al. (2020)
OPS1847	LM100 *glmS*::miniTn7T-aad9-T1-J23104-RBstd-*sfGFP*-DT16-T0)	This work
OPS1859	LM400 *glmS*::miniTn7T-aad9-T1-J23104-RBstd-*sfGFP*-DT16-T0	This work
OPS1871	LM100 *glmS*::miniTn7T-aad9-T1-J23104-RBstd-*gusA*-DT16-T0	This work
OPS1872	LM300 *glmS*::miniTn7T-aad9-T1-J23104-RBstd-*gusA*-DT16-T0	This work
OPS1873	LM400 *glmS*::miniTn7T-aad9-T1-J23104-RBstd-*gusA*-DT16-T0	This work
OPS1896	Rlv3841 *glmS*::miniTn7T-aad9-T1-J23104-RBstd-*gusA*-DT16-T0	This work
OPS1953	Rlv3841 *glmS*::miniTn7T-aad9-T1-J23104-RBstd-*sfGFP*-DT16-T0	This work
OPS2085	Rlv3841 *glmS*::miniTn7T-aad9-T1-J23104-RBstd-*celB*-DT16-T0	This work
OPS2107	Rlv3841 *ΔmotA*	This work
OPS2139	LM100 carrying pLMB712 (Rlv3841 pnodA::lux)	This work
OPS2140	LM300 carrying pLMB712 (Rlv3841 pnodA::lux)	This work
OPS2141	LM400 carrying pLMB712 (Rlv3841 pnodA::lux)	This work

OPS2163	Rlv3841 $\Delta fliG$	This work
OPS2234	Rlv3841 $\Delta flaA$	This work
OPS2086	LM100 *glmS*::miniTn7T-aad9-T1-J23104-RBstd-*celB*-DT16-T0	This work
OPS2088	LM400 *glmS*::miniTn7T-aad9-T1-J23104-RBstd-*celB*-DT16-T0	This work
OPS2447	OPS2163 *glmS*::miniTn7T-aad9-T1-J23104-RBstd-*gusA*-DT16-T0	This work
OPS2451	OPS2234 *glmS*::miniTn7T-aad9-T1-J23104-RBstd-*gusA*-DT16-T0	This work
OPS2526	OPS2163 carrying pLMB712 (Rlv3841 pnodA::lux)	This work
OPS2528	OPS2107 carrying pLMB712 (Rlv3841 pnodA::lux)	This work

Str^R: streptomycin resistance, Amp^R: ampicillin resistance, Tet^R: tetracyline resistance, Kan^R: kanamycin resistance, $Spec^R$: spectinomycin resistance

Table S3. Plasmids

Name	Description	Reference
pK18mobsacB	pK18 based mobilizable plasmid for double recombination in *Rhizobium*; suicide vector on sucrose; Kan^R	Schäfer et al. (1994)
pLMB712	pIJ11268 derivative; Rlv3841 *nodABC* promoter driving *luxCDABE*; Amp^R, Tet^R	Pini et al. (2017)
pOPS0857	pUC18T-miniTn7T-aad9-T1-J23104f-RBstd-*gusA*-DT16-T0; $Spec^R$	This work
pOPS0858	pUC18T-miniTn7T-aad9-T1-J23104-RBstd-*sfGFP*-DT16-T0; $Spec^R$	Guha et al. (2022)
pOPS1376	pUC18T-miniTn7T-aad9-T1-J123104-RBstd-*celB*-DT16-T0; Amp^R, $Spec^R$	This work
pOPS1412	pK18mobsacB derivative; containing flanking regions of Rlv3841 *motA*; F with oxp3773+oxp3774, R with oxp3775+oxp3776; Kan^R	This work

pOPS1422	pK18mobsacB derivative; containing flanking regions of Rlv3841 *fliG*; F with oxp3933+oxp3934, R with oxp3935+oxp3936; Kan^R	This work
pOPS1423	pK18mobsacB derivative; containing flanking regions of Rlv3841 *flaA*; F with oxp3921+oxp3922, R with oxp3923+oxp3924; Kan^R	This work
pRK2013	Helper plasmid for mobilisation of non-self-transmissible plasmids; Kan^R	Figurski & Helinski (1979)
pTNS3	Transposase plasmid for integration of mini-Tn7 cassettes; Amp^R	Choi and Schweizer (2006)

Str^R: streptomycin resistance, Amp^R: ampicillin resistance, Tet^R: tetracyline resistance, Kan^R: kanamycin resistance, $Spec^R$: spectinomycin resistance. F: upstream flanking region and R: downstream flanking region.

Table S4. Oligonucleotides

Name	Description	Sequence
M13F	Sense for sequencing of pK18mobSacB insertion	CACGACGTTGTAAAACGA
M13R	Antisense for sequencing of pK18mobSacB insertion	GGATAACAATTTCACACAGG
oxp2327	Sense for sequencing of miniTn7 insertion	GATGATCTTCTCGCTGCCGA
oxp3060	Antisense for sequencing of miniTn7 insertion	CACAGCATAACTGGACTGATTTC
oxp3773	Sense for PCR of *motA* F 1kb flanking region for cloning into pK18mobsacB	CGAATTCGAGCTCGGTACCCCGGAAGATCCAGGGAGAG
oxp3774	Antisense for PCR of *motA* F 1kb flanking region for cloning into pK18mobsacB	TGGGCTGACGATGACTATGAGCAATGCTTC
oxp3775	Sense for PCR of *motA* R 1kb flanking region for cloning into pK18mobsacB	TCATAGTCATCGTCAGCCCAATCTGAAC
oxp3776	Antisense for PCR of *motA* R 1kb flanking region for cloning into pK18mobsacB	GTCGACTCTAGAGGATCCCCAAAACCGCCGGACACTTTTG

oxp3919	Sense for mapping of *motA* in-frame mutation	GAAGTCTCGCCGCCAAAGG
oxp3920	Antisense for mapping of *motA* in-frame mutation	TCATGCTTCAAATCCCGCCC
oxp3933	Sense for PCR of *fliG* F 1kb flanking region for cloning into pK18mobsacB	CGAATTCGAGCTCGGTACCCTTGG CGATCATGCGCCGG
oxp3934	Antisense for PCR of *fliG* F 1kb flanking region for cloning into pK18mobsacB	TTAGACCATGCACGTCGAGACTGT TGACAAATC
oxp3935	Sense for PCR of *fliG* R 1kb flanking region for cloning into pK18mobsacB	TCTCGACGTGCATGGTCTAACCTC TCCG
oxp3936	Antisense for PCR of *fliG* R 1kb flanking region for cloning into pK18mobsacB	GTCGACTCTAGAGGATCCCCACTG CTCAAGACCAAGGTG
oxp3937	Sense for mapping of *fliG* in-frame mutation	GCGTGATCTTCGGCAAGGG
oxp3938	Antisense for mapping of *fliG* in-frame mutation	CGAACCGAGCCTTTCAGCC
oxp4055	Sense for sequencing of *motA* in-frame mutation	GATCCATCGCCGGTTTGTCG
oxp4056	Antisense for sequencing of *motA* in-frame mutation	GCAACTCGCCATTTCCCTCG
oxp4059	Sense for sequencing of *flaA* in-frame mutation	GCGAGATTGCCCTGGACATG
oxp4060	Antisense for sequencing of *flaA* in-frame mutation	TGGAGACCCTGGTTGATGCC
oxp4063	Sense for sequencing of *fliG* in-frame mutation	GCAAGGACCTAAAGCGCGTC
oxp4064	Antisense for sequencing of *fliG* in-frame mutation	TCGGCAGCGCTTTGAATTCC

Supplementary Information - Figures

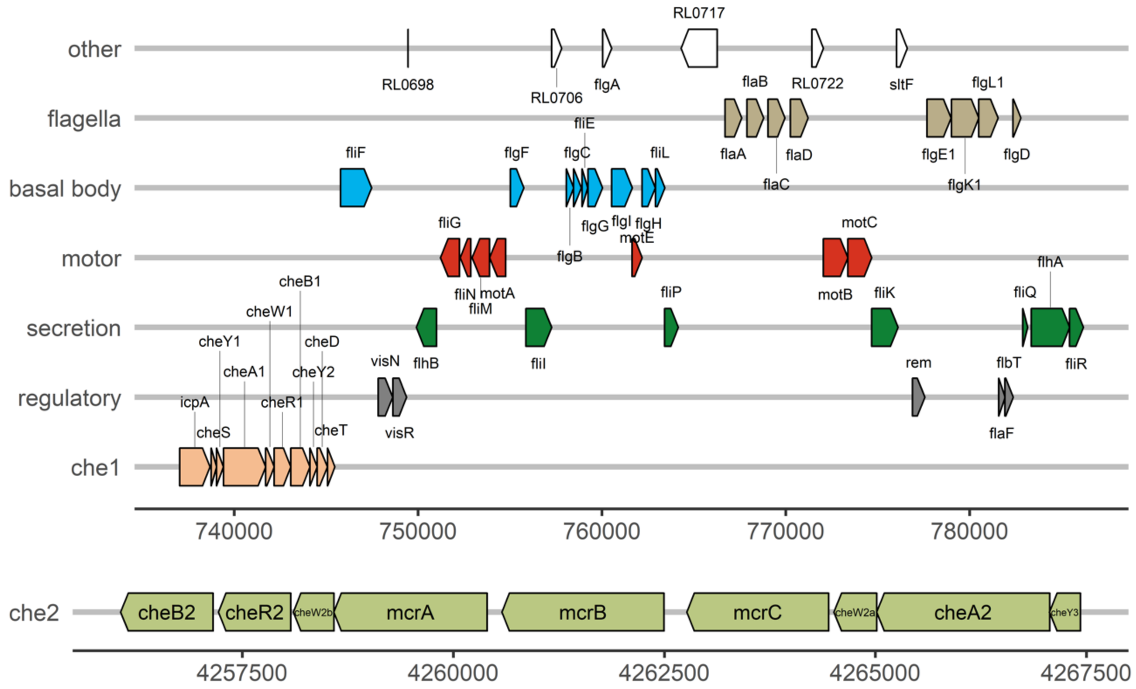

Figure S1. Rlv3841 motility and chemotaxis genes. The flagella and chemotaxis systems of Rlv3841 are present in only two clusters larger than three genes. The first (RL0685-736) contains genes encoding flagella, basal body, motor, type III secretion system, regulatory and Che1 proteins. The second (RL4028-36) contains genes encoding the Che2 proteins.

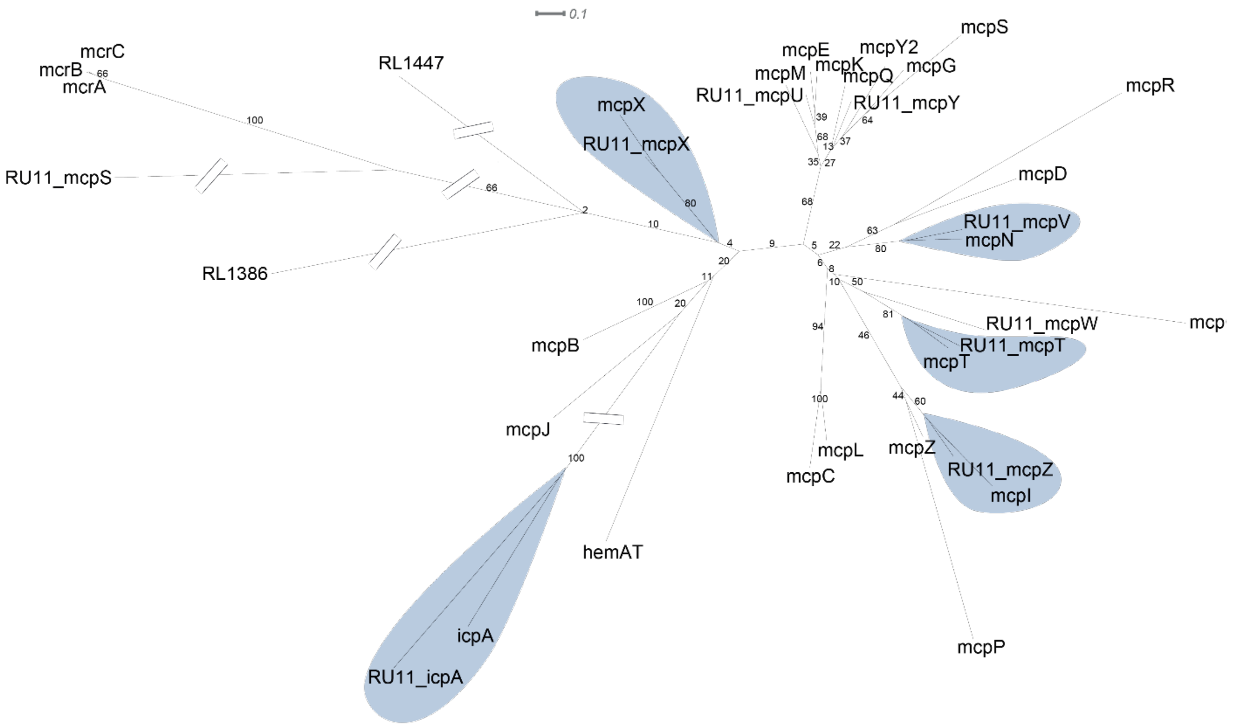

Figure S2. Maximum likelihood phylogenetic tree of chemoreceptor protein sequences from Rlv3841 and *S. meliloti* RU11/001 based on the MCP signal domain region. Orthologous groups of sequences are highlighted in grey. The tree was generated following a standard MCP analysis pipeline (Ortega and Zhulin, 2018) using the following tools: MAFFT 7.407, RAxML 8.2.12, HMMER 3.2.1, Jalview 2.10.5 and Dendroscope 3.5.10 (Waterhouse et al., 2009; Eddy, 2011; Huson and Scornavacca, 2012; Katoh and Standley, 2013; Stamatakis, 2014). Sequences from *R. leguminosarum* bv. *viciae* 3841 (NCBI:txid216596) and *S. meliloti* RU11/001 (NCBI:txid1401243) were obtained from the NCBI repository.

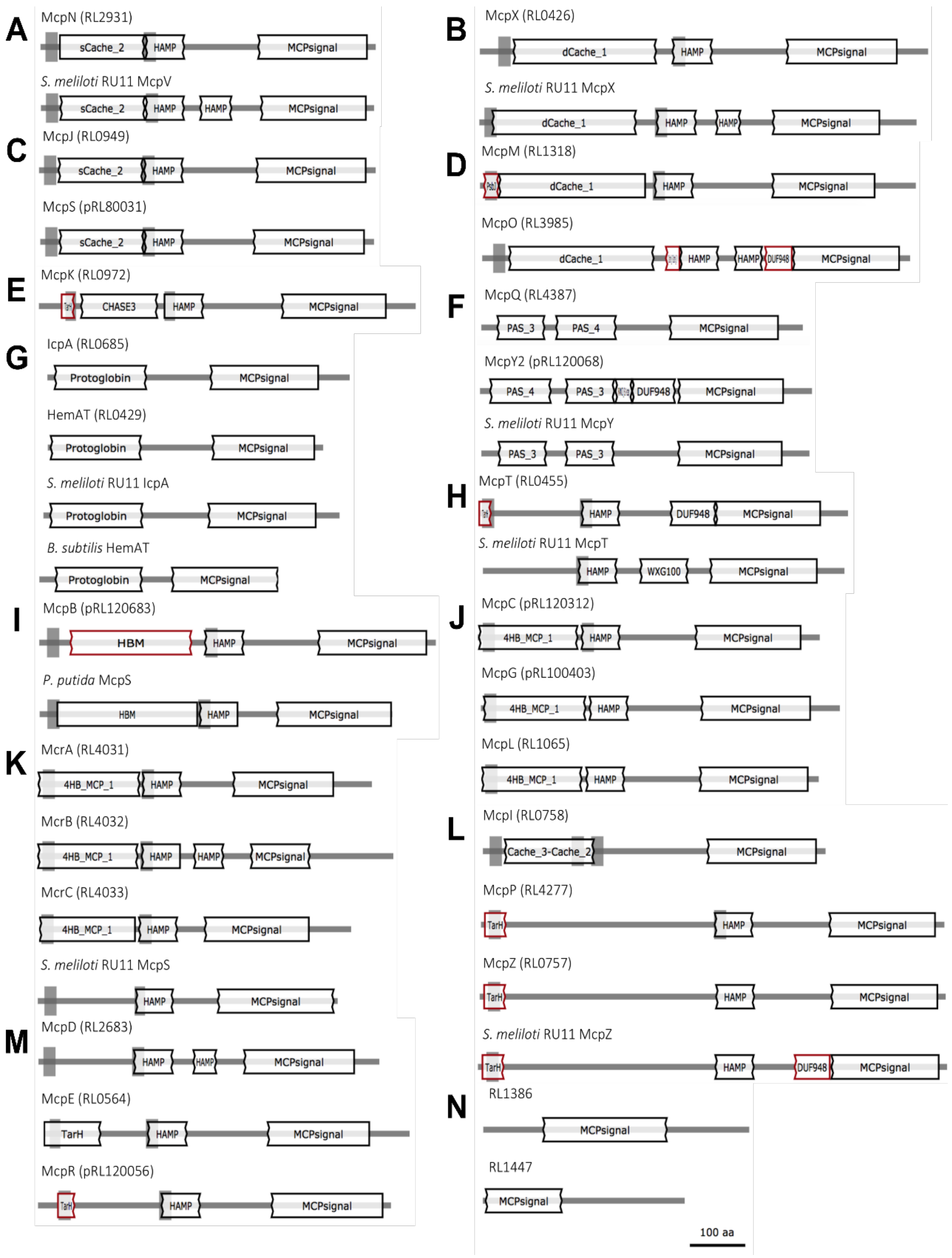
A
McpN (RL2931)
sCache_2 HAMP MCPsignal
S. meliloti RU11 McpV
sCache_2 HAMP HAMP MCPsignal
C
McpJ (RL0949)
sCache_2 HAMP MCPsignal
McpS (pRL80031)
sCache_2 HAMP MCPsignal
E
McpK (RL0972)
CHASE3 HAMP MCPsignal
G
IcpA (RL0685)
Protoglobin MCPsignal
HemAT (RL0429)
Protoglobin MCPsignal
S. meliloti RU11 IcpA
Protoglobin MCPsignal
B. subtilis HemAT
Protoglobin MCPsignal
I
McpB (pRL120683)
HBM HAMP MCPsignal
P. putida McpS
HBM HAMP MCPsignal
K
McrA (RL4031)
4HB_MCP_1 HAMP MCPsignal
McrB (RL4032)
4HB_MCP_1 HAMP HAMP MCPsignal
McrC (RL4033)
4HB_MCP_1 HAMP MCPsignal
S. meliloti RU11 McpS
HAMP MCPsignal
M
McpD (RL2683)
HAMP HAMP MCPsignal
McpE (RL0564)
TarH HAMP MCPsignal
McpR (pRL120056)
HAMP MCPsignal
B
McpX (RL0426)
dCache_1 HAMP MCPsignal
S. meliloti RU11 McpX
dCache_1 HAMP HAMP MCPsignal
D
McpM (RL1318)
dCache_1 HAMP MCPsignal
McpO (RL3985)
dCache_1 HAMP HAMP DUF948 MCPsignal
F
McpQ (RL4387)
PAS_3 PAS_4 MCPsignal
McpY2 (pRL120068)
PAS_4 PAS_3 DUF948 MCPsignal
S. meliloti RU11 McpY
PAS_3 PAS_3 MCPsignal
H
McpT (RL0455)
HAMP DUF948 MCPsignal
S. meliloti RU11 McpT
HAMP WXG100 MCPsignal
J
McpC (pRL120312)
4HB_MCP_1 HAMP MCPsignal
McpG (pRL100403)
4HB_MCP_1 HAMP MCPsignal
McpL (RL1065)
4HB_MCP_1 HAMP MCPsignal
L
McpI (RL0758)
Cache_3-Cache_2 MCPsignal
McpP (RL4277)
TarH HAMP MCPsignal
McpZ (RL0757)
TarH HAMP MCPsignal
S. meliloti RU11 McpZ
TarH HAMP DUF948 MCPsignal
N
RL1386
MCPsignal
RL1447
MCPsignal
100 aa

Figure S3. Chemoreceptor domain structures. Rlv3841 genes containing MCP signal domains compared to *S. meliloti* RU11/001, *Bacillus subtilis* and *Pseudomonas putida* chemoreceptors. (A) McpN (RL2931) and *S. meliloti* McpV domain structures. (B) McpX (RL0426) and *S. meliloti* McpX domain structures. (C) McpJ (RL0949) and McpS (pRL80031) domain structures. (D) McpM (RL1318) and McpO (RL3985) domain structures. (E) McpK (RL0972) domain structures. (F) McpQ (RL4387), McpY2 (pRL120068) and *S. meliloti* McpY domain structures. (G) IcpA (RL0685), HemAT (RL0429), *S. meliloti* IcpA and *B. subtilis* HemAT domain structures. (H) McpT (RL0455) and *S. meliloti* McpT domain structures. (I) McpB (pRL120683) and P. putida McpS domain structures. (J) McpC (pRL120312), McpG (pRL100403) and McpL (RL1065) domain structures. (K) McrA (RL4031), McrB (RL4032) and McrC (RL4033) domain structures. (L) McpI (RL0758), McpP (RL4277), McpZ (RL0757) and S. meliloti McpZ domain structures. (M) McpD (RL2683), McpE (RL0565) and McpR (pRL120056) domain structures. (N) RL1386 and RL1447 domain structures.

A

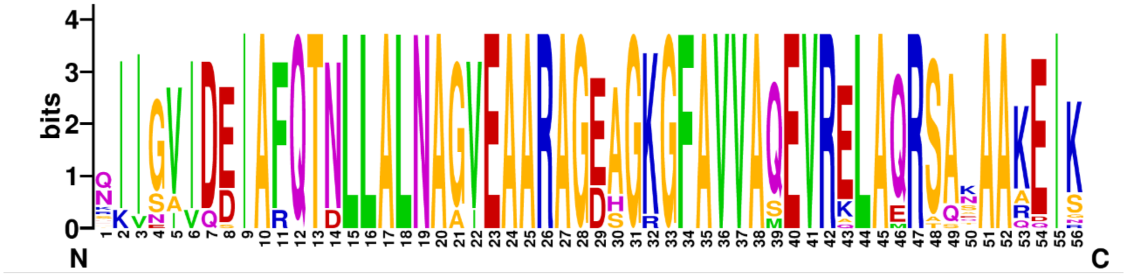

B

T E Q (Q) A A S L E (E) T A A S M E (Q) L T A T V

C

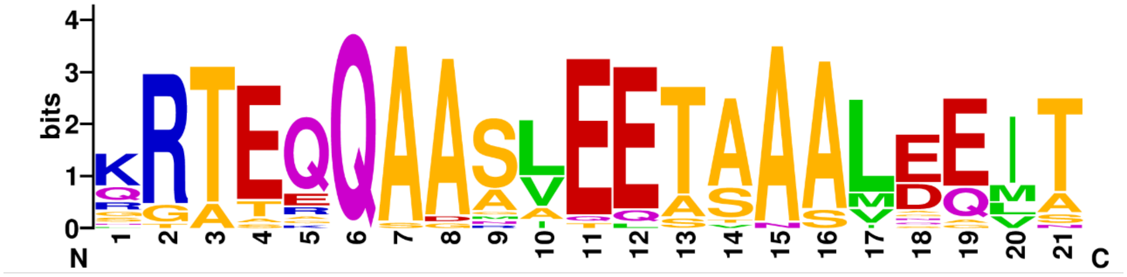

D

V T Q Q N A A L V E (E) S A A

E

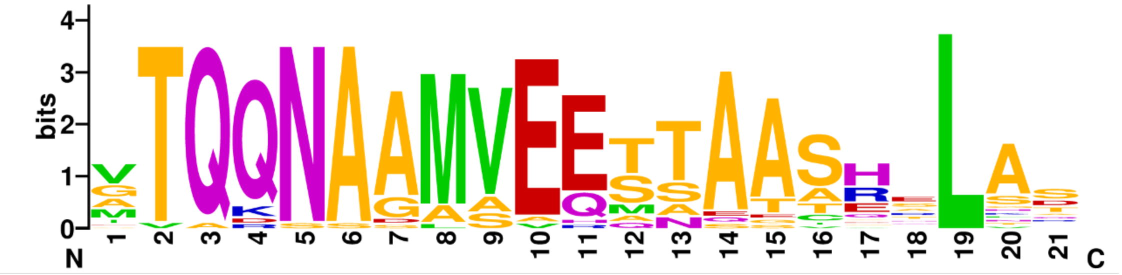

Figure S4. Chemoreceptor consensus motifs and logos. (A) Logo of the CheA binding site for all Rlv3841 chemoreceptors. (B) N-terminal methylation site in *Escherichia coli* Tsr. (C) Logo of the N-terminal methylation site for all R. leguminosarum chemoreceptors. (D) C-terminal methylation site in *E. coli* Tsr. (E) Logo of the C-terminal methylation site for all Rlv3841 chemoreceptors. Logos were generated using WebLogo.

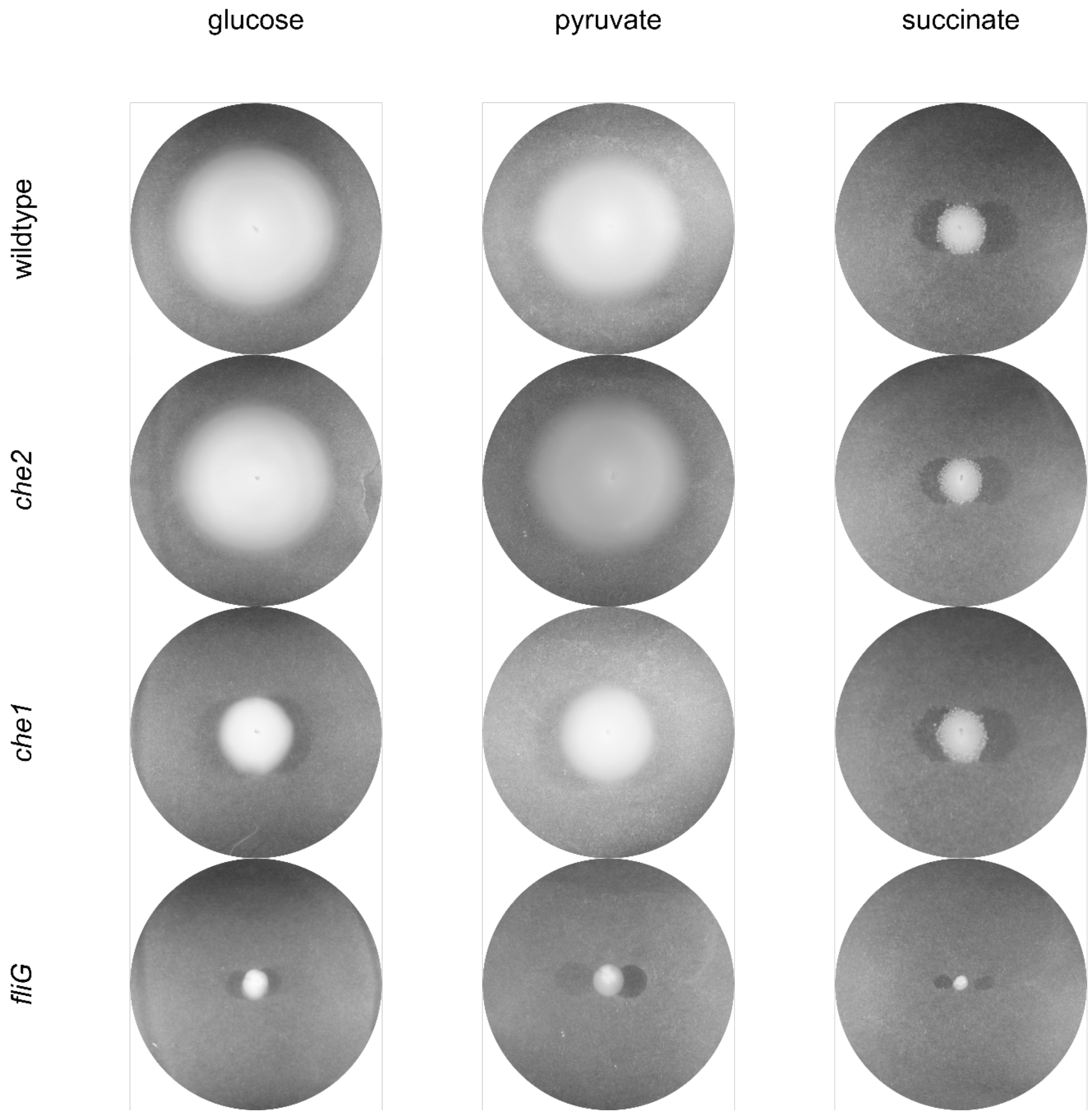

Figure S5: Swimming halos with high concentrations of carbon sources. Representative photos of soft-agar UMS plates showing swimming responses on UMS minimal media with 10 mM NH_4Cl and 10 mM glucose, 30 mM pyruvate and 20 mM succinate for Rlv3841 wildtype (images on the top row), Rlv3841 *che2* mutant LM400 (second row), Rlv3841 *che1* mutant LM100 (third row) and Rlv3841 *fliG* mutant OPS2163 (bottom row). Halos on succinate were small with cloudy spots.

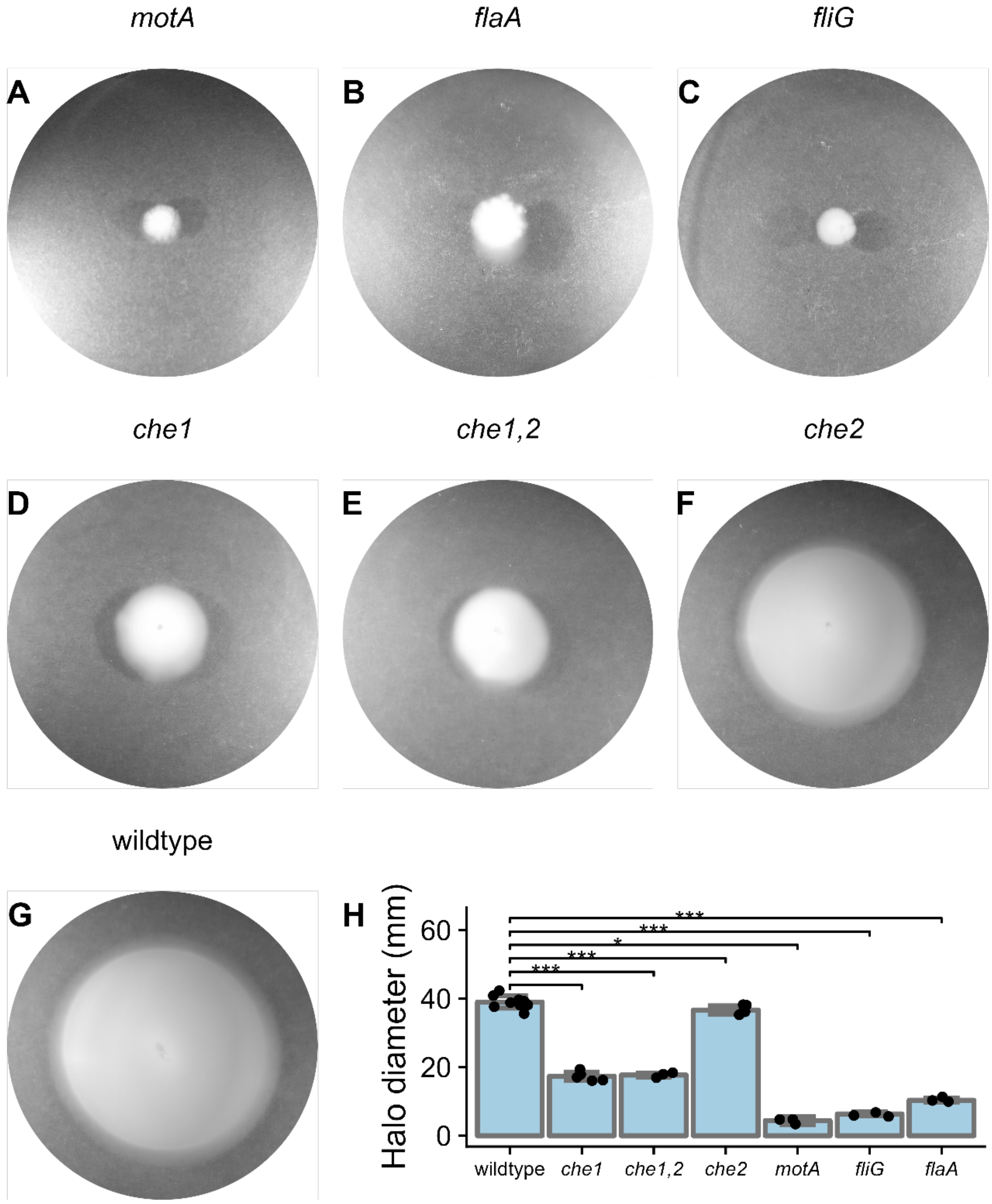

Figure S6. Photos of wildtype and mutant swimming halos. Representative photos of swimming halos on UMS semi-solid agar plates supplemented with 10 mM glucose and 10 mM NH_4Cl. Strains include *motA* mutant (OPS2107, A), *flaA* mutant (OPS2234, B), *fliG* mutant (OPS2163, C), *che1* cluster mutant (LM100, D), *che1,2* double cluster mutant (LM300, E), *che2* cluster mutant (LM400, F) and wildtype Rlv3841 (G). (H) Halo diameters of wildtype and mutants on swimming plates. Data was modelled with ANOVA and Dunnett's Post-hoc test comparing strains to wildtype, N=3-9. * P>0.05, *** P<0.001.

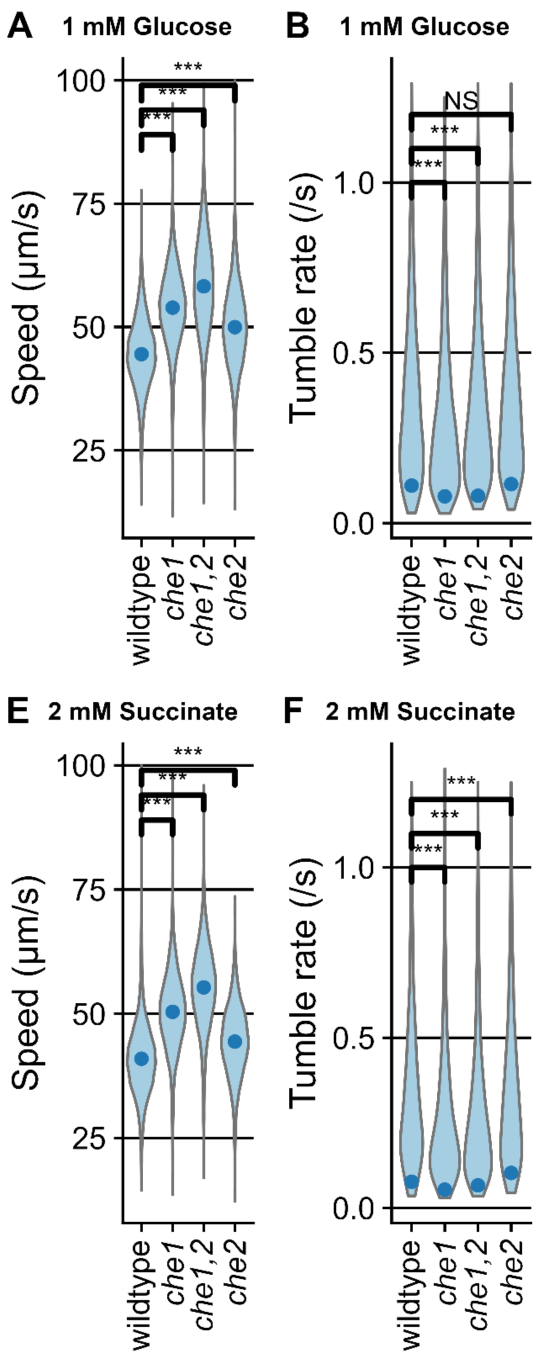

Figure S7. *R. leguminosarum* Rlv3841 swimming behaviour. Swimming tracking analysis in liquid cultures of UMS minimal media supplemented with 10 mM NH_4Cl as N source with 1 mM glucose (A, B), 3 mM pyruvate (C, D) and 2 mM succinate (E, F). Each track was analysed to detect non-tumble swimming speed (A, C, E) and tumble events (B, D, F). Data was modelled with ANOVA and Dunnett's Post-hoc test comparing strains to wildtype, with the tumble-rate analysis additionally weighting by track duration. Plots represent density of tracks with a given tumble rate or swimming speed, with the mean given as a point, with 10,000 to 40,000 tracks per group collated from three independent experiments each. NS (not significant), * P>0.05, *** P<0.001.

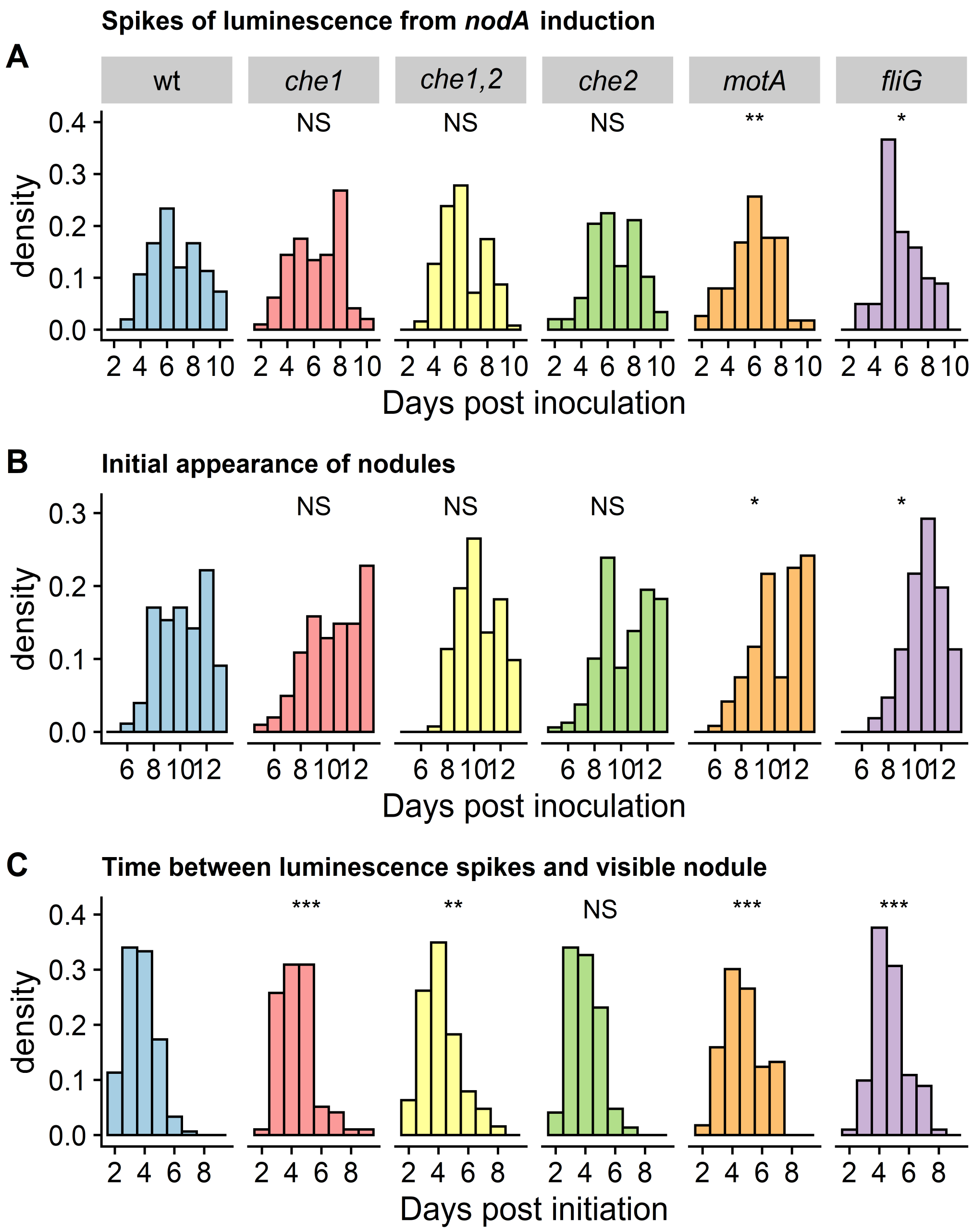

Figure S8: Nodulation dynamics assay. Tested strains were wildtype (LMB612), *che1* (OPS2139), *che1,2* (OPS2140), *che2* (OPS2141), *motA* (OPS2528) and *fliG* (OPS2526) harbouring a p*nodA-lux* promoter fusion. (A) Distribution of spikes of luminescence from *nodA* induction, only including spikes that resulted in nodule formation. (B) Distribution of initial appearance of nodules. (C) Distribution of times between the luminescence spike start and the corresponding nodules appearance. Distributions of each type modelled with a Gaussian distribution with strain parameter, significant in all types, $P<0.05$, ANOVA. Dunnett's Post-hoc test found significant differences in wildtype-strain comparisons with NS (not significant) $P>0.05$, * $P<0.05$, ** $P<0.01$, *** $P<0.001$. Histograms represent distributions of data from 5-8 independent growth plates each containing 5 plants, per strain.

References

Allaway, D., Schofield, N.A., Leonard, M.E., Gilardoni, L., Finan, T.M., and Poole, P.S. (2001) Use of differential fluorescence induction and optical trapping to isolate environmentally induced genes. *Environmental Microbiology* **3**: 397–406.

Boucher, C., Bergeron, B., De Bertalmio, M.B., and Dénarié, J. (1977) Introduction of Bacteriophage Mu into *Pseudomonas solanacearum* and *Rhizobium meliloti* using the R Factor RP4. *Microbiology* **98**: 253–263.

Choi, K.-H. and Schweizer, H.P. (2006) mini-Tn7 insertion in bacteria with single attTn7 sites: example *Pseudomonas aeruginosa*. *Nature Protocols* **1**: 153–161.

Eddy, S.R. (2011) Accelerated profile HMM searches. *PLoS Computational Biology* **7**:.

Figurski, D.H. and Helinski, D.R. (1979) Replication of an origin-containing derivative of plasmid RK2 dependent on a plasmid function provided in trans. *Proceedings of the National Academy of Sciences of the United States of America* **76**: 1648–1652.

Guha, S., Molla, F., Sarkar, M., Ibañez, F., Fabra, A., and DasGupta, M. (2022) Nod factor-independent 'crack-entry' symbiosis in dalbergoid legume Arachis hypogaea. *Environmental Microbiology* **24**: 2732–2746.

Hanahan, D. (1983) Studies on transformation of *Escherichia coli* with plasmids. *Journal of Molecular Biology* **166**: 557–580.

Hmelo, L.R., Borlee, B.R., Almblad, H., Love, M.E., Randall, T.E., Tseng, B.S., et al. (2015) Precision-engineering the *Pseudomonas aeruginosa* genome with two-step allelic exchange. *Nature Protocols* **10**: 1820–1841.

Huson, D.H. and Scornavacca, C. (2012) Dendroscope 3: An interactive tool for rooted phylogenetic trees and networks. *Systematic Biology* **61**: 1061–1067.

Johnston, A.W.B. and Beringer, J.E. (1975) Identification of the *Rhizobium Strains* in Pea Root Nodules Using Genetic Markers. *Journal of General Microbiology* **87**: 343–350.

Katoh, K. and Standley, D.M. (2013) MAFFT multiple sequence alignment software version 7: Improvements in performance and usability. *Molecular Biology and Evolution* **30**: 772–780.

Miller, L.D., Yost, C.K., Hynes, M.F., and Alexandre, G. (2007) The major chemotaxis gene cluster of *Rhizobium leguminosarum* bv. *viciae* is essential for competitive nodulation. *Molecular Microbiology* **63**: 348–362.

Ortega, D.R. and Zhulin, I.B. (2018) Phylogenetic and Protein Sequence Analysis of Bacterial Chemoreceptors. In *Bacterial Chemosensing: Methods and Protocols*. Manson, M.D. (ed). New York, NY: Springer New York, pp. 373–385.

Pédelacq, J.-D., Cabantous, S., Tran, T., Terwilliger, T.C., and Waldo, G.S. (2006) Engineering and characterization of a superfolder green fluorescent protein. *Nature Biotechnology* **24**: 79–88.

Pini, F., East, A.K., Appia-Ayme, C., Tomek, J., Karunakaran, R., Mendoza-Suárez, M., et al. (2017) Bacterial Biosensors for *in vivo* Spatiotemporal Mapping of Root Secretion. *Plant Physiol* **174**: 1289–1306.

Prell, J., Mulley, G., Haufe, F., White, J.P., Williams, A., Karunakaran, R., et al. (2012) The PTSNtr system globally regulates ATP-dependent transporters in *Rhizobium leguminosarum*. *Molecular Microbiology* **84**: 117–129.

Sánchez-Cañizares, C., Prell, J., Pini, F., Rutten, P., Kraxner, K., Wynands, B., et al. (2020) Global control of bacterial nitrogen and carbon metabolism by a PTSNtr-regulated switch. *Proceedings of the National Academy of Sciences* **117**: 10234–10245.

Schäfer, A., Tauch, A., Jäger, W., Kalinowski, J., Thierbach, G., and Pühler, A. (1994) Small mobilizable multi-purpose cloning vectors derived from the Escherichia coli plasmids pK18 and pK19: selection of defined deletions in the chromosome of *Corynebacterium glutamicum*. *Gene* **145**: 69–73.

Stamatakis, A. (2014) RAxML version 8: A tool for phylogenetic analysis and post-analysis of large phylogenies. *Bioinformatics* **30**: 1312–1313.

Thoma, S. and Schobert, M. (2009) An improved *Escherichia coli* donor strain for diparental mating. *FEMS Microbiol Lett* **294**: 127–132.

Waterhouse, A.M., Procter, J.B., Martin, D.M.A., Clamp, M., and Barton, G.J. (2009) Jalview Version 2-A multiple sequence alignment editor and analysis workbench. *Bioinformatics* **25**: 1189–1191.
